# Exosomal miRNAs from Prostate Cancer Impair Osteoblast Function in Mice

**DOI:** 10.3390/ijms23031285

**Published:** 2022-01-24

**Authors:** Giulia Furesi, Antonio Miguel de Jesus Domingues, Dimitra Alexopoulou, Andreas Dahl, Matthias Hackl, Johannes R. Schmidt, Stefan Kalkhof, Thomas Kurth, Hanna Taipaleenmäki, Stefanie Conrad, Christine Hofbauer, Martina Rauner, Lorenz C. Hofbauer

**Affiliations:** 1 Department of Medicine III & Center for Healthy Aging, Technical University of Dresden, 01307 Dresden, Germany; furesi@wustl.edu (G.F.); Stefanie.Conrad@uniklinikum-dresden.de (S.C.); martina.rauner@uniklinikum-dresden.de (M.R.); 2 Max Planck Institute of Molecular Cell Biology and Genetics, 01307 Dresden, Germany; amjdomingues@gmail.com; 3 DRESDEN-Concept Genome Center, DFG NGS Competence Center, c/o Center for Molecular and Cellular Bioengineering (CMCB), Technical University of Dresden, 01307 Dresden, Germany; dimitra.alexopoulou@tu-dresden.de (D.A.); andreas.dahl@tu-dresden.de (A.D.); 4 TAmiRNA GmbH, 1110 Vienna, Austria; matthias.hackl@tamirna.com; 5 Department of Preclinical Development and Validation, Fraunhofer Institute for Cell Therapy and Immunology IZI, 04103 Leipzig, Germany; johannes.schmidt@izi.fraunhofer.de (J.R.S.); Stefan.Kalkhof@hs-coburg.de (S.K.); 6 Institute of Bioanalysis, University of Applied Sciences and Arts of Coburg, 96450 Coburg, Germany; 7 Center for Molecular and Cellular Bioengineering (CMCB), Technology Platform, EM and Histology Facility, TU Dresden, 01307 Dresden, Germany; Thomas.Kurth@tu-dresden.de; 8 Institute of Musculoskeletal Medicine (IMM), Musculoskeletal University Center Munich (MUM), University Hospital, LMU Munich, 82152 Planegg-Martinsried, Germany; Hanna.Taipaleenmaeki@med.uni-muenchen.de; 9 Molecular Skeletal Biology Laboratory, Department of Trauma and Orthopedic Surgery, University Medical Center Hamburg-Eppendorf, 20246 Hamburg, Germany; 10 National Center for Tumor Diseases, Technical University of Dresden, 01307 Dresden, Germany; christine.hofbauer@uniklinikum-dresden.de

**Keywords:** bone metastases, prostate cancer, osteoprogenitors, extracellular vesicles, miRNA

## Abstract

Prostate cancer (PCa) is the most frequent malignancy in older men with a high propensity for bone metastases. Characteristically, PCa causes osteosclerotic lesions as a result of disrupted bone remodeling. Extracellular vesicles (EVs) participate in PCa progression by conditioning the pre-metastatic niche. However, how EVs mediate the cross-talk between PCa cells and osteoprogenitors in the bone microenvironment remains poorly understood. We found that EVs derived from murine PCa cell line RM1-BM increased metabolic activity, vitality, and cell proliferation of osteoblast precursors by >60%, while significantly impairing mineral deposition (−37%). The latter was further confirmed in two complementary in vivo models of ossification. Accordingly, gene and protein set enrichments of osteoprogenitors exposed to EVs displayed significant downregulation of osteogenic markers and upregulation of proinflammatory factors. Additionally, transcriptomic profiling of PCa-EVs revealed the abundance of three microRNAs, miR-26a-5p, miR-27a-3p, and miR-30e-5p involved in the suppression of BMP-2-induced osteogenesis in vivo, suggesting the critical role of these EV-derived miRNAs in PCa-mediated suppression of osteoblast activity. Taken together, our results indicate the importance of EV cargo in cancer-bone cross-talk in vitro and in vivo and suggest that exosomal miRNAs may contribute to the onset of osteosclerotic bone lesions in PCa.

## 1. Introduction

Skeletal metastases are a frequent complication of prostate cancer (PCa) and a major cause of morbidity and mortality in patients with advanced disease [1,2]. Proliferation of PCa cells within the bone promotes localized bone turnover, resulting in primarily osteosclerotic lesions with a concomitant osteolytic component [3]. These skeletal alterations markedly impact life quality as they are associated with pathological fractures, severe pain, and shorter survival. Establishment of PCa metastasis to a distant site depends on a complex cascade of events, including invasion, colonization, and proliferation of tumor cells within a foreign microenvironment [4]. As first postulated by Stephen Paget [5], the factors that determine organ specificity of metastatic tumors are derived from both the seed (tumor cells) and the soil (host environment). Of note, cross-talks between bone and PCa severely disrupt physiological bone remodeling, with a substantial alteration of host cell behavior. Therein, tumor cells support their growth and guarantee a high local adaptation and survival. Moreover, emerging evidence indicates that primary PCa cells can alter the bone microenvironment even before the metastatic spread has occurred, suggesting that a pre-metastatic niche is educated by tumor cells through mechanisms that are not fully understood [6,7].

Over the past years, extracellular vesicles (EVs) have been identified as critical mediators of tumor progression and metastatic evolution, primarily via their ability to pre-educate the metastatic site [8]. EVs are a heterogeneous group of membranous structures secreted by the cells into the extracellular space [9]. Their central feature is to transfer active molecules (e.g., nucleic acids, proteins, and lipids) to recipient cells, thereby influencing their behavior [10,11]. Thus, EVs from the primary tumor can selectively modify distant sites and create a milieu that fosters tumor growth [12,13,14]. For instance, recent studies showed that PCa-EVs instruct bone-resident cells towards a pro-tumorigenic cell type by influencing their endogenous transcript abundance and enhancing metastatic organotropism [15,16]. To date, exosomal microRNAs (miRNAs) have been identified as critical molecular tools involved in tumor progression and metastasis [17]. MiRNAs are small non-coding RNAs that bind to the 3′UTRs of target messenger RNAs (mRNAs) and repress gene expression by promoting mRNA degradation or inhibiting translation. Within the metastatic bone environment, PCa-derived exosomal miRNAs have been shown to modulate bone-resident cell activity by controlling their maturation, proliferation, and differentiation [18,19]. These findings suggest that bone remodeling can be regulated by tumor-derived miRNAs during metastasis. However, detailed insights into this interaction are still largely unknown.

Here, we investigated the impact of EVs secreted by the murine PCa cells RM1-BM on osteoblastic function. Education of osteoprogenitors with PCa-EVs inhibited osteogenesis in vitro and in vivo, induced a proinflammatory osteoblast phenotype, and increased their ability to enhance tumor metabolic activity and migration. As a potential mechanism, we identified miR-26a-5p, miR-27a-3p, and miR-30e-5p, which are highly enriched in PCa-EVs, as mediators that suppress osteoblast activity. Thus, our results suggest that uptake of PCa-derived exosomal miRNAs by osteoblasts and their precursors may represent the transfer signal to alter osteoblast function in PCa bone metastasis.

## 2. Materials and Methods

### 2.1. Murine Primary Osteoblast Culture

Hind legs of 10–12-week-old male C57BL/6J wild-type mice (obtained from Janvier Laboratories, Le Genest-Saint-Isle, France) were harvested, and mesenchymal stromal cells were acquired by flushing the bone marrow. Cells were cultured to 80% confluence in growth medium consisting of Dulbecco’s Modified Eagle’s Medium (DMEM; Thermo Fisher Scientific, Schwerte, Germany), 10% fetal bovine serum (FBS; Biochrom, Berlin, Germany), 1% penicillin/streptomycin (P/S; Invitrogen, Schwerte, Germany) in a humidified atmosphere of 95% air and 5% CO_2_ at 37 °C, and then differentiated for seven days into osteoprogenitors by adding 100 µM ascorbate phosphate and 5 mM β-glycerol phosphate (both from Sigma Aldrich, Munich, Germany) to the growth medium.

### 2.2. Murine Prostate Cancer Cell Line RM1-BM

The murine bone metastatic prostate carcinoma cell line RM1-BM-derived from C57BL/6J mice was kindly provided by Professor Pamela J. Russel, Oncology Research Centre, NSW, Australia [20]. Cells are androgen receptor-positive; yet display androgen independence, thereby reflecting the castration-resistant prostate cancer phenotype in humans. Cells were cultured in DMEM supplemented with 10% FBS and 1% P/S in a humidified atmosphere of 95% air and 5% CO_2_ at 37 °C and were chosen to have a syngeneic cell culture system.

### 2.3. Inhibition of miRNAs in RM1-BM Cells

To knock down the target miRNAs, RM1-BM were transfected with 50nM anti-miR-17-5p, anti-miR-24-3p, anti-miR-26a-5p, anti-miR-27a-3p, and anti-miR-30e-5p (Thermo Fisher Scientific, Schwerte, Germany) using DharmaFECT1 (Thermo Fisher Scientific, Schwerte, Germany) according to the manufacturer’s instructions. To exclude non-specific effects, a non-targeting anti-miRNA at the same concentration was used as control. Due to the high proliferation rate of RM1-BM cells, anti-miRNAs had to be added every 24 h for two consecutive days to achieve a sufficient knockdown. After 48 h, the supernatant was harvested, and EVs were isolated according to our standardized protocol. Finally, miRNAs knockdown in RM1-BM cells was validated based on the reduction in gene expression using qPCR.

### 2.4. EV Preparation

EVs were isolated from murine prostate cancer cell line RM1-BM. Upon reaching 90% confluence, cells were washed with Hank’s balanced salt solution (HBSS) (Thermo Fisher Scientific, Schwerte, Germany) and starved in serum-free DMEM to prevent FBS-derived EV contamination. After 48 h, culture medium was harvested, and EVs were isolated by serial centrifugations. Briefly, supernatant was first centrifuged at 400× *g* for 10 min, 2000× *g* for 30 min, and 10,000× *g* for 45 min to exclude, respectively, floated cells, cell debris, and large vesicles. Supernatant was then filtered through a 0.22 µm filter and concentrated (40-fold) to a final volume of 1 mL using 10,000 molecular weight cut-off Vivaspin 20 centrifugal concentrators (Sartorius, Göttingen, Germany). Finally, EVs were pelleted by ultracentrifugation at 100,000× *g* for 4 h (Beckman, SW 32 Ti Swinging-Bucket Rotor Krefeld, Germany). The final pellet was resuspended in HBSS, and the concentration was determined via BiCinchonic Acid (BCA) assay using the Pierce BCA Protein Assay Kit (Thermo Fisher Scientific, Schwerte, Germany) according to the manufacturer’s protocol.

### 2.5. Nanoparticle Tracking Analysis

To examine EV size distribution and particle concentration, nanoparticle tracking analysis (NTA) was performed using a ZetaView instrument (Particle Metrix, Inning am Ammersee, Germany). Acquisition parameters were set to a minimum brightness of 20, a maximum size of 200 pixels, and a minimum size of 5 pixels. Samples were diluted 1:2000 in particle-free PBS to achieve a particle count in the range of 100 to 200 vesicles per visual field. Concentration, mean, and median diameters of the samples were calculated using the ZetaView software 8.05.05 SP2. Data acquisition thresholds were set to a shutter of 70, a sensitivity of 80%, and a frame rate of 30 frames per second.

### 2.6. Transmission Electron Microscopy

Isolated RM1-BM-EVs (5–10 μL) were pipetted into a 300-mesh copper grid covered with a thin carbon-coated and glow discharged formvar film and incubated for 10 min to allow EVs to sediment and adhere to the film. The liquid was removed, and the EVs were stained with 1% uranyl acetate in water for about 20 sec. Uranyl acetate was removed slowly with filter paper, and the grid was air-dried before inspection with a Jeol JEM-1400Plus transmission electron microscope (Jeol, Freising, Germany) at 80 kV acceleration voltage.

### 2.7. Western Blot Analysis

EVs were lysed in a buffer containing 20 mM Tris/HCl pH 7.4, 1% sodium dodecyl sulfate (SDS), and a protease inhibitor (complete mini, Roche, Mannheim, Germany). The protein concentration was assessed using the BCA assay (Thermo Fisher Scientific, Schwerte, Germany). Twenty µg of heat-denatured protein was loaded onto a 10% gel, separated, and transferred onto a 0.2 μm nitrocellulose membrane (Whatman, Dassel, Germany). After blocking for 1 h with 5% non-fat dry milk in Tris-buffered saline with 1% Tween-20 (TBS-T), membranes were incubated overnight with the following primary antibodies: CD63 (1:200, Abcam, Cambridge, UK; ab217345), CD9 (1:200, Abcam, Cambridge, UK; ab92726), GAPDH (1:2000, Hytest, Turku, Finland; 5G4). Thereafter, membranes were incubated with the appropriate HRP conjugated secondary antibodies (1:1000) for 1 h at room temperature (RT). Finally, membranes were washed with TBS-T and incubated with an ECL substrate (Thermo Fisher Scientific, Schwerte, Germany). The proteins were visualized using the MF-ChemiBIS 3.2 bioimaging system (Biostep, Burkhardtsdorf, Germany).

### 2.8. EV Labeling

PCa-EVs were labeled with the red fluorescent lipophilic dye PKH26 (Sigma-Aldrich, Munich, Germany) according to the manufacturer’s instructions. Briefly, EVs were resuspended in 1 mL Dilution C, and 2× Dye Solution (4 × 10^−6^ M) was prepared by adding 4 μL of the PKH26 to 1 mL of Diluent C. The two solutions were mixed and incubated for 5 min at RT. The stain reaction was stopped by adding media with 20% EVs-depleted FCS. PKH-labeled EVs were finally pelleted by ultracentrifugation. Supernatant was collected and used as control to exclude any non-EV-associated fluorescence dye.

### 2.9. Fluorescence Microscopy

Primary mesenchymal stromal cells were differentiated into osteoblast precursors for seven days and treated with labeled EVs (20 µg/mL) or media deriving from the labeling washing steps for up to 24 h. After the incubation time, cells were washed with phosphate-buffered saline (PBS) and fixed with 4% paraformaldehyde (PFA). Cells were permeabilized with 0.1% Triton-X-100 for 30 min. F-actin was labeled by adding Alexa-Fluor-488 conjugated phalloidin (Invitrogen, Schwerte, Germany), and nuclei were stained with 2.5 µg/mL of 4′,6-diamidin-2-phenylindol (DAPI; Sigma-Aldrich, Munich, Germany). Cells were embedded in mounting medium, and the uptake was quantified using the microscope Axio Imager M1 (Zeiss, Jena, Germany).

### 2.10. Flow Cytometric Analysis

Internalization of EVs was further analyzed by flow cytometry. Seven days differentiated osteoblasts were exposed to EVs-PHK26+ (20 µg/mL) or media deriving from the labeling washing steps for 1, 4, 8, and 24 h. Cells were then trypsinized, centrifuged for 5 min at 500× *g*, and resuspended in 1 mL buffer (PBS with 5% FCS). Thereafter, cells were transferred to flow cytometry tubes and stained with DAPI (1 μg/mL). The accumulation of PCa-EVs in the osteoblast precursors was determined by the fluorescence emission of PKH26 using the BD LSR II flow cytometer and the BD Diva software (BD Biosciences, Heidelberg, Germany).

### 2.11. Cell Metabolic Activity Assay

Seven days differentiated osteoblasts were treated with increasing concentrations of RM1-BM-EVs (20, 50, 100 µg/mL) for 48 and 72 h, respectively. Cellular ATP content was then assessed using fluorescence CellTiter-Blue^®^ reagent (Promega, Mannheim, Germany) according to the manufacturer’s protocol. To further investigate the impact of pre-treated osteoblast precursors on RM1-BM cells, an indirect co-culture was performed. Supernatant from osteoprogenitors treated for 48 h with PCa-EVs (100 μg/mL) was collected. RM1-BM cells were seeded into a 96-well plate in depleted culture medium (DMEM 10% EVs-free FBS and 1% P/S) at a density of 1 × 10^4^/well. After 24 h, 50% of EV-treated osteoprogenitor supernatant was added. Untreated RM1-BM or RM1-BM treated with normal osteoprogenitor supernatant were used as control. RM1-BM metabolic activity was measured by performing a CellTiter-Blue^®^ assay as previously indicated.

### 2.12. Cell Vitality Assay

To determine the vitality of osteoprogenitors after EV treatment, a crystal violet staining was performed. Seven days differentiated osteoblasts were washed with PBS and fixed using 10% PFA for 15 min at RT. Cells were washed with double-distilled water (ddH20) and stained with crystal violet solution (0.02% in 2% ethanol) for 20 min at RT. Stained cells were intensively rinsed with ddH20 and dried afterward. The crystal violet dye was eluted with 10% SDS upon shaking. Absorbance was detected at 595 nm using the FluoStar Omega (BMG Labtech, Ortenberg, Germany).

### 2.13. Evaluation of Osteogenesis In Vitro

Osteoprogenitors were differentiated and treated with PCa-EVs every three days for ten days. Afterward, cells were fixed in 10% PFA for 15 min and stained with 1% Alizarin red S solution, pH 5.5 (Sigma-Aldrich, Munich, Germany) for 30 min at RT. Excessive staining was removed by repeated washing steps using distilled water. The amount of calcium was determined in pixels using the image processing program ImageJ software (v 1.5.1).

### 2.14. Wound Healing Assay

Supernatant from osteoprogenitors treated with PCa-EVs (100 μg/mL) for 48 h was collected. RM1-BM cells were seeded into a 6-well plate with culture medium (DMEM 5% FBS and 1% P/S) at a density of 1 × 10^6^/well and incubated at 37 °C and 5% CO_2_ until they reached 100% of confluence. The confluent monolayer was then scraped in a straight line to create a scratch with a 200 μL sterile pipette tip. Culture medium was discarded, and the detached cells were removed by washing the wells with PBS. 50% of EV-treated osteoprogenitor supernatant was added. Untreated RM1-BM or RM1-BM treated with EVs-free osteoblast supernatant were used as control. Wound healing was observed and captured by optical microscope (Zeiss, Jena, Germany) with 10× magnification at 0, 4, and 8 h. The percentage of covered areas was analyzed using the ImageJ software (v 1.5.1).

### 2.15. RNA Isolation, Reverse Transcription, and Real-Time-PCR

Total RNA from osteoprogenitors and RM1-BM cells was isolated with the High Pure RNA Isolation Kit (Roche, Mannheim, Germany) or miRNeasy Mini Kit (Qiagen, Hilden, Germany) according to the manufacturer’s protocol. The RNA quality and quantity were quantified using the NanoDrop spectrophotometer (Peqlab, Erlangen, Germany). To analyze the expression of osteogenic markers, RNAs were reverse-transcribed using M-MLV RT RNase (H-) Point Mutant (Promega, Mannheim, Germany) followed by SYBR Green-based quantitative real-time PCR (qPCR) according to established protocols (ABI7500 Fast; Applied Biosystems, Darmstadt, Germany). The primer sequences were: *Alp* S: CTACTTGTGTGGCGTGAAGG, *Alp* AS: CTGGTGGCATCTCGTTATCC; *Bmp-2* S: CGCTCCACAAACGAGAAAAG, *Bmp-2* AS: CAGTCATTCCACCCCACATC; *Gapdh* S: AAGGTCATCCCAGAGCTGAA, *Gapdh* AS: CTGCTTCACCACCTTCTTGA; *Ocn* S: GCGCTCTGTCTCTCTGACCT, *Ocn* AS: ACCTTATTGCCCTCCTGCTT; *Opg* S: CCTTGCCCTGACCACTCTTA, *Opg* AS: ACACTGGGCTGCAATACACA; *Osx* S: CTTCCCAATCCTATTTGCCGTTT, *Osx* AS: CGGCCAGGTTACTAACACCAATCT; and *Runx2* S: AAATGCCTCCGCTGTTATGAA, *Runx2* AS: GCTCCGGCCCACAAATCT. qPCR conditions were: 95 °C for 2 min followed by 40 cycles with 95 °C for 15 sec and 60 °C for 1 min. Melting curves were assessed by the following scheme: 95 °C for 15 sec, 60 °C for 1 min, and 95 °C for 30 sec.

To analyze the expression of miR-17-5p, miR-24-3p, miR-26a-5p, miR-27a-3p, and miR-30e-5p, RNAs were reverse-transcribed using miScript II RT Kit (Qiagen, Hilden, Germany), and relative miRNA expression levels were determined by qPCR using miScript SYBR Green PCR Kit (Qiagen, Hilden, Germany). The following primers were used: Hs_RNU6-2_11, Mm_miR-17_1, Mm_miR-24_1, Mm_miR-26a_2, Mm_miR-27a_1, Mm_miR-30e_2 (MiScript Primer Assay; Qiagen, Hilden, Germany). PCR conditions were assessed according to the manufacturer’s protocol. Results were calculated based on the ΔΔCT method and are depicted in x-fold change normalized to the housekeeping gene *Gapdh* (for osteogenic genes) and RNU6-2 (for miRNAs).

### 2.16. Next-Generation Sequencing and Data Analysis

Total RNA was extracted from osteoprogenitors untreated and treated with 100 µg/mL PCa-EVs for 48 h using High Pure RNA isolation kit (Roche, Mannheim, Germany). RNA quality was assessed using the Agilent Bioanalyzer. mRNA was isolated from 300 ng total RNA by poly-dT enrichment using the NEBNext Poly(A) mRNA Magnetic Isolation Module according to the manufacturer’s instructions. Samples were eluted in 11.5 µL of a mixture of NEBNext First-Strand Synthesis Buffer and Random Primers and then directly subjected to the workflow for strand-specific RNA-Seq library preparation (Ultra Directional RNA Library Prep, NEB). For ligation, custom adaptors were used (Adaptor-Oligo 1: 5′-ACA CTC TTT CCC TAC ACG ACG CTC TTC CGA TCT-3′, Adaptor-Oligo 2: 5′-P-GAT CGG AAG AGC ACA CGT CTG AAC TCC AGT CAC-3′). After ligation, adapters were depleted by an XP bead purification adding the beads solution in a ratio of 1:0.9. Dual unique indexing was done during the following PCR enrichment (12 cycles, 65 °C) using custom amplification primers carrying the same sequence for i7 and i5 index (Primer 1: AAT GAT ACG GCG ACC ACC GAG ATC TAC AC NNNNNNNN ACA TCT TTC CCT ACA CGA CGC TCT TCC GAT CT, Primer 2: CAA GCA GAA GAC GGC ATA CGA GAT NNNNNNNN GTG ACT GGA GTT CAG ACG TGT GCT CTT CCG ATC T). After two more XP bead purifications (1:0.9), libraries were quantified using the Fragment Analyzer. For Illumina flowcell production, samples were equimolarly pooled and sequenced 75 bp single read on an Illumina NextSeq 500.

After sequencing, RNA-SeQC [21] was used to perform basic quality control on the resulting reads. As an additional control, library diversity was assessed by redundancy investigation in the mapped reads. Alignment of the short reads to the mouse transcriptome (mm10) was performed using GSNAP v2018-07-04 [22]. A table with counts per gene was obtained by running featureCounts v1.6.3 [23] on the uniquely mapped reads using Ensembl Genes annotations (v.92). Normalization of the raw read counts based on the library size and testing for differential expression between pre-osteoprogenitors treated and untreated with PCa-EVs was performed with the DESeq2 R package v. 1.22.2 [24]. Sample to sample Euclidean distance, as well as Pearson’s correlation coefficient (r), were computed based on the normalized gene expression level to explore the correlation between biological replicates and different libraries. For testing differential expression, the count data were fitted to the negative binomial distribution. The *p*-values for the statistical significance of the fold change were adjusted for multiple testing with the Benjamini-Hochberg correction, accepting a maximum of 10% false discoveries (*p*_adj_ ≤ 0.1).

Gene ontology analyses were performed with Cytoscape v3.7.2 and the ClueGO plugin. Only significantly (*p* < 0.05) up or down-regulated genes were fed into the analyses. Gene Set Enrichment Analysis (GSEA) was carried out using the Broad Institute GSEA software “GseaPreRanked” tool (n_perm_ = 1000, set_min = 5, set_max = 500, scoring_scheme = weighted) to analyze a list of 18,106 non-redundant gene symbols ranked by their log2 fold-change of expression between osteoprogenitors with or without EVs treatment. In total 58 gene sets were used for the analysis, including 50 Hallmark gene sets, three osteoblast-specific gene sets from Park et al. and Zaidi et al., and five other gene sets from Sanjuan-Pla et al. [25,26,27].

### 2.17. Proteomics of PCa-EVs and PCa-EV Stimulated Osteoprogenitor Cells

Proteins were extracted from RM1-BM cells and RM1-BM-derived EVs, as well as osteoprogenitor cells untreated or treated with 100 µg/mL tumor-EVs for 48 h using buffer containing 20 mM Tris/HCl pH 7.4, 1% sodium dodecyl sulfate (SDS), and a protease inhibitor (complete mini, Roche, Mannheim, Germany). The protein concentration was assessed using the BCA assay (Thermo Fisher Scientific, Schwerte, Germany). For filter-aided sample preparation (FASP) on a centrifugal filter unit (Vivacon 500, 10K MWCO, Sartorius, Göttingen, Germany), the protein lysate was depleted from the detergent and amine containing buffer components. Therefore, urea buffer (8 M urea in 100 mM triethylammonium bicarbonate, pH 8.5) was added to the corresponding volume of 15 µg of each protein lysate to a final volume of 500 µL, and samples were centrifuged at 14,000× *g* for 30 min. This applies to all following centrifugation steps, if not indicated otherwise. Reduction was carried out using 10 mM tris(2-carboxyethyl)phosphine (TCEP) in urea buffer and reaction was stopped by centrifugation. Next, denatured proteins were thiol-alkylated using 55 mM iodoacetamide (IAA) in urea buffer for 30 min incubation in the dark. The filter units were centrifuged and subsequently, the proteins were washed twice with urea buffer. Ultimately, proteins were subjected to proteolytic digestion in a digestion solution (1 M urea in 100 mM triethylammonium bicarbonate buffer) containing 300 ng Trypsin/Lys-C protease mix (protein/enzyme ratio = 50/1) for 16 h at 37 °C. Digested peptides were collected by ultrafiltration and centrifugation for 14,000× *g* for 60 min. The filter device was rinsed with 0.5 M NaCl and centrifuged. The filtrate was acidified by adding formic acid to final concentration of 1% (v/v). Collected peptides were desalted using Pierce Peptide Desalting Spin Columns (Thermo Fischer Scientific, Rockford, IL, USA) and their concentration was determined by NanoDrop analysis and absorbance at 205 nm (Thermo Fischer Scientific, Rockford, IL, USA). 4 µg of peptides of each sample was labeled by 10-plex tandem mass tags (Thermo Fischer Scientific, Rockford, IL, USA) according to manufactures protocol and pooled. The pooled sample was fractionated to eight fractions and re-pooled to four fractions using Pierce High pH Peptide Fractionation Kit (Thermo Fischer Scientific, Rockford, IL, USA). Gained samples were evaporated and reconditioned in 0.1% formic acid.

A total of 500 ng of peptides were injected to Easy-nLC 1200 coupled to a Q Exactive HF mass spectrometer (both Thermo Fisher Scientific, Rockford, IL, USA). Peptides were separated on 20 cm analytical HPLC column (75 µm ID Pico Tip fused silica emitter, New Objective, Littleton, CO, USA) in-house packed using ReproSil-Pur C18-AQ 1.9-μm silica beads (Dr. Maisch GmbH, Ammerbuch, Germany) applying a 90 min multistep gradient from 10% solvent B to 90% solvent B at a constant flow rate of 200 nL/min. The mobile phases were prepared as 0.1% formic acid in water (solvent A) and 80% ACN, supplemented with 0.1% formic acid in water (solvent B). Eluting peptides were ionized by electrospray ionization at 3.2 kV. Data were acquired in data dependent mode and controlled by XCalibur software (version 2.9). Survey scans were acquired in orbitrap mass analyzer in scan range 300–1650 m/z with a mass resolution of 60,000, AGT target of 3 × 10^6^, and a maximum injection time of 25 ms. Top 10 most abundant precursor ions were selected for isolation (window of 0.7 m/z) and fragmentation by Higher-energy collisional dissociation (normalized collision energy: 33). For MS2 scans in orbitrap mass analyzer, AGT target and maximum injection time were set to 1 × 10^5^ and 110 ms, respectively. A dynamic exclusion for MS2 scans was set to 30 s. Proteomics raw data were analyzed using MaxQuant (version 1.6.3.3.) with the integrated algorithm Andromeda [28]. The spectra were matched to the murine reference protein database (UniprotKB, release 2020_02). The mass errors were limited to 20 ppm (first search and fragment ions) and 4.5 ppm (main search), respectively. A maximum of two missed cleavages was allowed for trypsin set to specific mode. Carbamidomethylation was set as fixed modification whereas oxidation of methionine was allowed as variable modification. A minimum of one unique peptide was required for protein identification. FDR was controlled to 0.01 for peptide and protein assignment in target-decoy approach. Reporter ion-based quantification and normalization for lot specific impurities at MS2-level was applied to all samples.

Sample analysis was performed in Perseus (version 1.6) [29]. Potential contaminants and reverse entries were filtered from identifications. Normalized reporter ion intensities were log_2_-transformed and median subtracted per quantification channel. To identify EV-enriched proteins, normalized reporter ion intensities of RM1-BM-released EVs were compared to RM1-BM cells. A fold change cutoff was determined empirically: All identified proteins were referenced to Top 100 protein list of ExoCarta database (release 29 July 2015) [30]. The enrichment of top 100 ExoCarta proteins among all proteins at a certain log_2_ fold change cutoff was calculated by Fisher’s exact test. The log_2_ fold change was reduced until exceeding a *p*-value of 0.05 first time at log_2_ fold change < 0.7. Thus, all proteins with log_2_ fold change > 0.7 and Benjamini–Hochberg adjusted *p*-value (FDR) < 0.01 were assigned as enriched in PCa EVs. Differential protein abundance of PCa EV-treated to non-treated osteoprogenitors was statistically determined by Student’s *t*-test (unpaired, two-sided). Proteins possessing *p*-value < 0.05 and absolute log_2_ fold change > 0.1 of EV treated compared to untreated cells were assigned as differentially abundant (DAP). GSEA was carried out according to next generation sequencing data analysis.

### 2.18. Small RNA Deep Sequencing of Prostate Cancer-Derived EVs

To investigate miRNAs cargo of RM1-BM-EVs, total RNA was isolated using the miRNeasy miRNA isolation mini kit according to the manufacturer’s protocol. Small RNA libraries were prepared using the NEXTFLEX^®^ Small RNA-Seq Kit v3 (PerkinElmer, Rodgau, Germany). Briefly, the NEXTflex 3’ and NEXTflex 5’ 4N Adapter were diluted 1:2, and the amplification was performed with 18 cycles. The amplified libraries were purified with AMPure XP beads (Beckman Coulter, Krefeld, Germany) by using a 2:1 bead:sample ratio. The purified libraries were quantified with Fragment Analyzer (AATI) and sequenced on a NextSeq500 system. Mapping steps were performed with bowtie v1.2.2 [31] and miRDeep2 v2.0.1.2 [32], whereas reads were mapped first against the genomic reference GRCm38.p6 provided by Ensembl [33], allowing for two mismatches and subsequently miRBase v22.1 [34], filtered for miRNAs of mmu only, allowing for one mismatch. For a general RNA composition overview, non-miRNA mapped reads were mapped against RNAcentral [35] and then assigned to various RNA species of interest.

### 2.19. Integration of miRNA Expression and Differential Gene Expression in Osteoprogenitors

To integrate the miRNA expression in tumor-derived EVs and gene expression changes in osteoprogenitors treated with RM1-BM EVs, the validated miRNA-target interactions from miRTarBase were used [36]. The following data processing steps were taken: the top 50 most expressed miRNAs (RPM) in tumor EVs were selected. These were merged with the miRTarBase miRNA-target information and with the differential expressed genes in osteoprogenitors treated with EVs. Finally, only genes differentially expressed in the treated osteoprogenitors were kept [37].

### 2.20. In Vivo Experiments

All animal procedures were approved by the Institutional Animal Care Committee and the Landesdirektion Sachsen (TVV 17/2020). 10-week-old C57BL/6J male mice were obtained from Janvier Laboratories and fed a standard diet with water ad libitum and were kept in groups of 4 animals per cage. Mice were exposed to a 12 h light/dark cycle and an air-conditioned room at 23 °C (no specific pathogen-free room). Enrichment was provided in form of cardboard houses and bedding material. Mice were randomly assigned to treatment groups, and the subsequent analyses were performed in a blinded fashion.

Ectopic ossification using alginate beads: Alginic acid sodium salt (Sigma-Aldrich, Munich, Germany) was dissolved in calcium-free PBS, and osteoprogenitors were resuspended in the solution in combination or not with PCa-EVs (100 µg/mL). As control, alginate beads with no cells were used. The suspension was then dripped into 100 mM calcium chloride buffer, pH 7.4 (Sigma-Aldrich, Munich, Germany). After solidification, the loaded beads were implanted subcutaneously on the flanks of the mice. Recovery phase of the mice was monitored under an infrared lamp for the next 2 h. After 5 weeks, animals were euthanized, and the ossification of the beads was measured using µCT (Scanco Medical, Brüttisellen, Switzerland).

Calvarial defect model: The osteogenic potency of PCa-EVs was evaluated using a mouse calvarial defect model. Recombinant human bone morphogenetic protein 2 (BMP-2) (1 µg; Gibco^®^, Darmstadt, Germany), PCa-EVs (100 µg/mL), BMP-2 + PCa-EVs, or BMP-2 + PCa-EVs inhibited for the target miRNAs were allowed to absorb into sterile gelatin sponges (B. Braun Surgical, Melsungen, Germany) for 1 min. A critical calvarial defect was generated on the parietal bone of 10-week-old male wild-type mice using a biopsy punch (Hager & Meisinger, Neuss, Germany; 3.5 mm of diameter). The gelatin sponge was then placed on the defect. Mice were sacrificed after 21 days, and calvariae were fixed in formaldehyde (4%) for µCT analysis.

### 2.21. Micro-Computed Tomography

Bone defect healing and formation of ectopic bone were analyzed using the vivaCT40 (Scanco Medical, Brüttisellen, Switzerland). The calvariae were imaged ex vivo at a resolution of 10.5 μm with X-ray energy of 70 kVp, 114 mA, and an integration time of 200 ms. Both alginate beads and half of the calvaria were contoured to produce a 3D reconstruction. Bone volume (BV) of the beads was analyzed using established analysis protocols from Scanco Medical, while the calvarial healed area was measured as a percentage using the image processing program ImageJ (v 1.5.1).

### 2.22. Statistical Analysis

All data are presented as the mean ± standard deviation (SD). Statistical evaluations were performed with GraphPad Prism 7.0 Software (GraphPad, San Diego, CA, USA) using one-way analysis of variance (ANOVA) followed by post hoc test or unpaired Student’s *t*-test for single group comparison. The values were considered significant at *p* < 0.05. The results are representative of more than three independently performed experiments.

## 3. Results

### 3.1. Characterization of PCa-EVs

**Figure 1:**
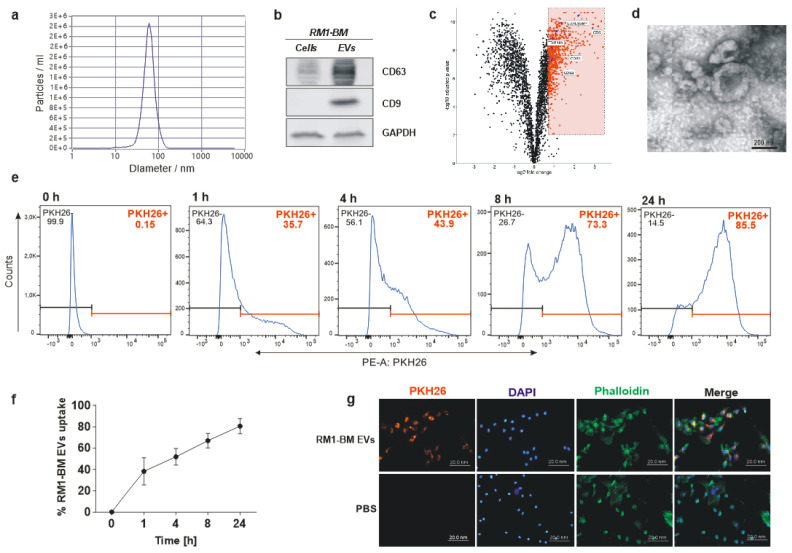
Characterization of RM1-BM EVs and integration into osteoblastic target cells. (**a**) Determination of size distribution and relative concentration of EVs using nanoparticle tracking analyzer (NTA). (**b**) Western blot analysis of CD9 and CD63 with extracts from RM1-BM cells and freshly isolated EVs. (**c**) Determination of EV-enriched proteins by proteomics represented as volcano plot of log_2_ fold change (x-axis) and statistical significance (y-axis) of protein abundances in RM1-BM-released EVs vs. RM1-BM cells. red = statistically enriched proteins and blue = Top 100 ExoCarta entries among enriched proteins. (**d**) Representative transmission electron microscopy image of PCa-derived EVs. Scale bar: 200 nm. (**e**) Flow cytometric analysis and (**f**) percentage of PKH26-labeled EV uptake by osteoprogenitors after 0, 1, 4, 8, and 24 h of exposure. Data are presented as mean ± SD. (**g**) Representative immunofluorescence images of seven-day differentiated osteoprogenitors with labeled RM1-BM EVs or EVs-free medium (CO) for 24 h. Labeled EVs (PKH26) = red; F-actin cytoskeleton (phalloidin) = green; nuclei (DAPI) = blue. Scale bars = 200 µm. Results are representative of four independent experiments.

To assess the involvement of tumor-derived EVs in bone-PCa cell communication, we first isolated and characterized EVs from the murine prostate cancer cell line RM1-BM according to the MISEV2018 guidelines provided by the International Society of Extracellular Vesicles [38]. Media was harvested after 48 h of conditioning under serum starvation, and EVs were collected using an established ultracentrifugation method. Examination of the size distribution by NTA revealed a high concentration of purified EVs (2.5 × 10^6^ particles/mL on average) in the size range of ~70–150 nm (Figure 1a), demonstrating an enrichment in the exosome population, albeit with the presence of non-exosomal microvesicles and apoptotic bodies. To further confirm the bona fide isolation of EVs, we used Western blot analysis to test for the presence of two of the most commonly-reported exosomal markers, the transmembrane proteins CD9 and CD63. PCa-EVs showed enrichment of those proteins compared to whole cell lysates (Figure 1b). Additionally, proteins enriched in RM1-BM-released EVs compared to originating cells were determined by proteomics. Among the 823 EV-enriched proteins, 31 are listed in top 100 ExoCarta entries indicating a statistical overrepresentation (Fisher’s *t*-test, *p* < 0.05) and including typical exosomal markers CD9, CD63, CD81, TSG101, and LGALS3BP (Figure 1c, Table S1). Further, gene ontology term enrichment analysis identified 311 of those proteins included in term “extracellular vesicle” resulting in a statistical over-representation (*p* = 1.6 × 10^−46^). Last, the vesicular appearance of the particles was investigated by transmission electron microscopy, which confirmed membrane integrity and the characteristic ovoid shape of EVs (Figure 1d). Taken together, the results demonstrate an enrichment of exosomes by the described preparation.

### 3.2. Uptake of PCa-EVs by Osteoblastic Recipient Cells

Because bone metastasis resulting from PCa tend to be osteosclerotic rather than osteolytic, we postulated a potential role of PCa-EVs as mediators of signal transfer between cancer cells and osteoblasts. To test our hypothesis, we first examined if and how fast osteoprogenitors take up PCa-EVs in cell culture. Seven days differentiated osteoprogenitors derived from bone marrow stromal cells of WT mice were exposed for 1, 4, 8, and 24 h to PKH26-labelled EVs at a concentration of 20 μg/mL (Figure 1e). To exclude unspecific signals, cells were treated with cultured media derived from the washing steps after EV-labeling and used as a negative control. After the respective incubation time, osteoprogenitors were washed and analyzed by flow cytometry and fluorescence microscopy. PCa-EVs were detected in the target cells as early as after 1 h of incubation, indicating rapid uptake of PCa-EVs by osteoprogenitors. After 24 h, nearly all osteoprogenitors contained tumor-derived EVs (Figure 1f,g), confirming the spontaneous internalization of EVs, allowing intercellular communication.

### 3.3. PCa-EVs Alter Osteoprogenitor Behavior In Vitro

**Figure 2:**
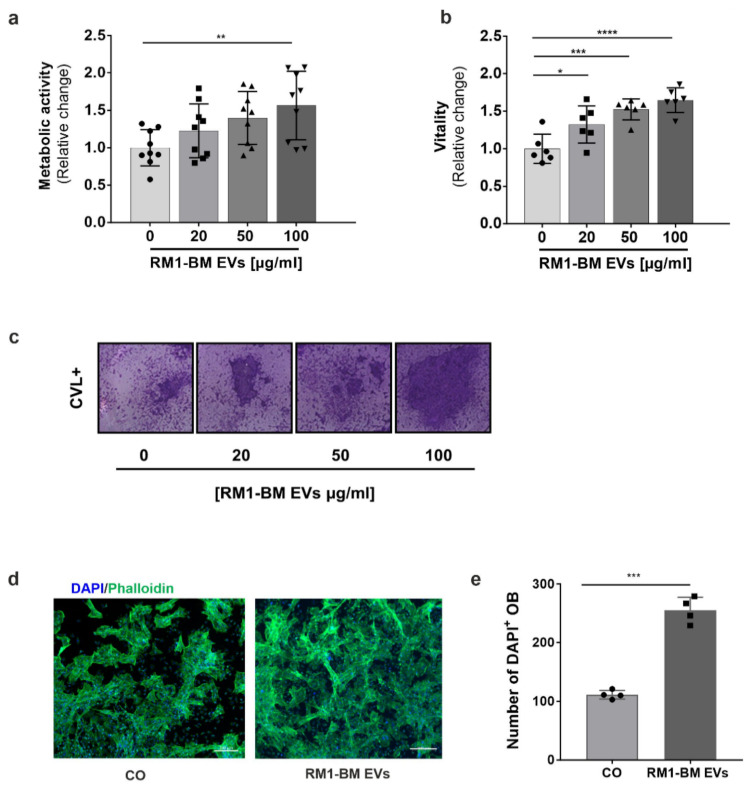
RM1-BM EVs enhance osteoblastic activity. Seven-day differentiated osteoprogenitors were treated with increasing concentration of RM1-BM EVs for 48 h. (**a**) Osteoprogenitor metabolic activity was evaluated based on CellTiter Blue assay. (**b**) Crystal violet assay was performed to determine cellular vitality (**a**,**b**) *n* = 6–9). (**c**) Representative image of Crystal violet staining. (**d**) Representative fluorescence microscopy pictures of osteoblast precursors treated with 100 µg/mL of RM1-BM EVs for 48 h and stained with Phalloidin (F-actin = green) and DAPI (nuclei = blue). Scale bar: 200 µm. (**e**) Number of DAPI-stained osteoprogenitor nuclei (*n* = 4). Statistical analyses were performed by one-way ANOVA with Tukey’s post hoc test (**a**,**b**) or unpaired Student’s *t*-test (**e**). Data are presented as mean ± SD. Statistical significance is denoted: * *p* < 0.05; ** *p* < 0.01; *** *p* < 0.001; **** *p* < 0.0001 vs. untreated cells. Circles: control, squares: 20 µg/ml RM1-BM EVs, triangle: 50 µg/ml RM1-BM EVs, inverse triangle: 100 µg/ml RM1-BM EVs.

Based on the substantial role of osteoblasts in the progression of PCa in bone, we next assessed whether PCa-EVs could modify the physiological behavior of osteoprogenitors in vitro. Assessment of metabolic activity demonstrated a dose-dependent increase in osteoprogenitors treated with RM1-BM EVs (20, 50, 100 µg/mL) for 48 h, as compared to the untreated cells (Figure 2a). Accordingly, osteoprogenitor vitality (Figure 2b,c) was enhanced after PCa-EVs exposure, reaching the highest level at 100 µg/mL of vesicle concentration. Further, fluorescence microscopy of phalloidin/DAPI stained osteoprogenitors showed no significant changes of the actin cytoskeleton after 48 h of EVs treatment (100 μg/mL) (Figure 2d). However, the number of DAPI+ cells was higher (+56%) in osteoprogenitors treated with PCa-EVs compared to HBSS-treated controls (Figure 2e), suggesting an increased cell proliferation after PCa-EVs exposure.

### 3.4. PCa-Derived EVs Impair Osteoblast Mineralization

**Figure 3:**
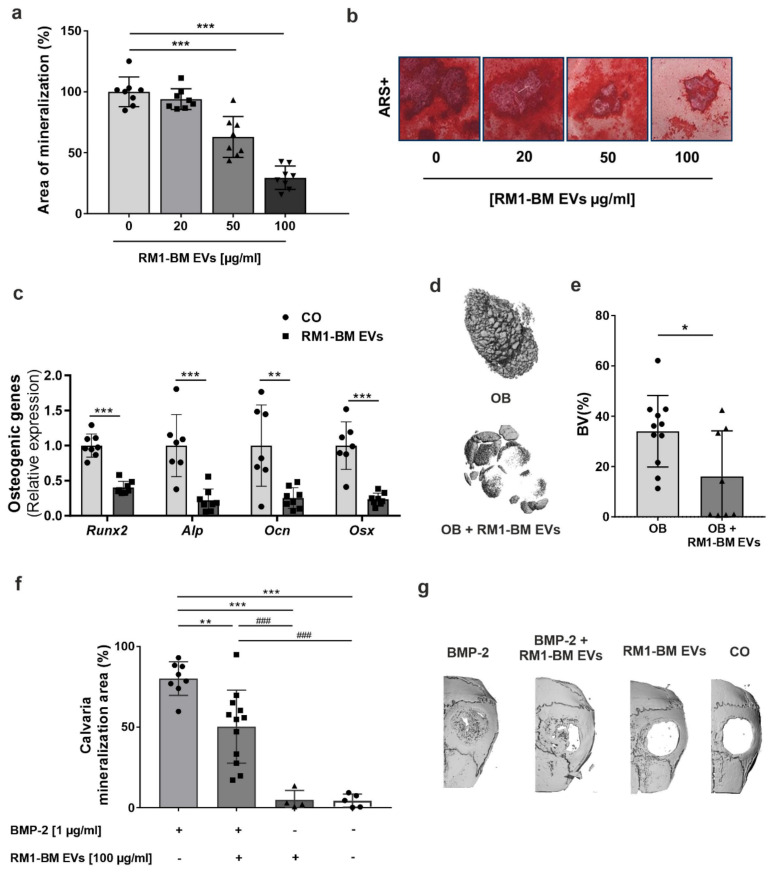
RM1-BM EVs impair osteoblast differentiation and mineralization. (**a**) Quantification of the Alizarin Red S staining of bone marrow stromal cell-derived osteoblasts treated for 10 days with increasing concentration of RM1-BM EVs. Untreated cells were used as control (*n* = 8). Circles: control, squares: 20 µg/ml RM1-BM EVs, triangle: 50 µg/ml RM1-BM EVs, inverse triangle: 100 µg/ml RM1-BM EVs. (**b**) Representative image of Alizarin Red staining. (**c**) qPCR analysis of osteoblast markers in osteoprogenitors after 48 h of treatment with 100 μg/mL RM1-BM EVs (*n* = 7–8). Circles: control, squares: 20 µg/ml RM1-BM EVs. (**d**) Representative 3D pictures and (**e**) quantification of bone volume by micro-computed tomography (μCT) analysis of ectopic ossification in alginate beads (*n* = 8–11). Circles: control, triangle: 50 µg/ml RM1-BM EVs. (**f**) Quantitative analyses of newly-formed bone after 21 days of sponges implantation (*n* = 4–13). Circles in grey bar: BMP-2, squares: BMP2+RM1-BM EVs, triangle: RM1-BM EVs, circles in white bar: control. (**g**) Three-dimensional (3D) μCT pictures of bone formation at the critical-sized calvarial defects. Statistical analysis was performed by one-way ANOVA with Tukey’s post hoc test (**a**,**f**) or unpaired Student’s *t*-test (**c**,**d**). Data are presented as mean ± SD. Statistical significance is denoted: * *p* < 0.05; ** *p* < 0.01; *** *p* < 0.001 vs. untreated cells (**a**,**c**,**e**); ** *p* < 0.01; *** *p* < 0.001 vs. BMP-2 treated group and ### *p* < 0.001 vs. BMP-2+ PCa-EVs treated group (**f**).

To evaluate the effect of tumor EVs on osteoblast function, we quantified calcium deposits in mineralized osteoblasts by assessing Alizarin Red staining. Murine primary osteoprogenitors were cultured in osteogenic induction medium for 10 days and simultaneously treated every other day with an increasing concentration (20, 50, 100 µg/mL) of RM1-BM EVs. Exposure to PCa-EVs dose-dependently reduced the osteoblastic mineral deposition as compared to the untreated cells, in which the mineralization was readily apparent (Figure 3a,b). In agreement with the mineralization assay, mRNA levels of the osteoblastic markers *Runx2,* *Alp, Ocn*, and *Osx* were significantly decreased by ~60, 80, 75, and 80%, respectively (Figure 3c), indicating that osteoblastogenesis was suppressed by RM1-BM EVs. 

To determine whether PCa-EVs also inhibit osteoblast activity in vivo, a mouse model of ectopic bone formation was used. Seven days differentiated osteoblasts were encapsulated into alginate beads with or without RM1-BM EVs. The microbeads were subcutaneously implanted into opposite flanks of WT mice for 5 weeks. Analysis of ectopic ossification by μCT revealed a two-fold decrease in bone formation in beads containing RM1-BM EVs and osteoprogenitors compared to unexposed osteoprogenitors (Figure 3d,e). Correspondingly, inhibition of bone mineralization by PCa-EVs was also observed using a complementary mouse calvarial defect model, in which bone formation was induced using BMP-2. To that end, BMP-2 (1 µg), RM1-BM EVs (100 µg/mL), or a combination of both were loaded into gelatin sponges before implantation into the calvaria defect. After 21 days, the BMP-2 treatment led to an almost complete closure of the bone defect (+78%, Figure 3f,g), whereas the combination of BMP-2 and PCa-EVs led to a significantly reduced rate of newly-formed bone (30% reduction) and EVs-only did not induce any skeletal regeneration (+4.8%) (Figure 3f,g). The latter result was also observed in mice implanted with unloaded sponges, excluding interference associated with the chemical composition of the sponges. Altogether, these data indicate that PCa-EVs inhibit osteoblast function in vitro and in vivo and thus suggest that they may be involved in the pre-metastatic dysregulation of bone remodeling.

### 3.5. PCa-Derived EVs Support Inflammatory Response in Osteoprogenitors and Diminish Gene Expression of Bone Mineralization Factors

**Figure 4:**
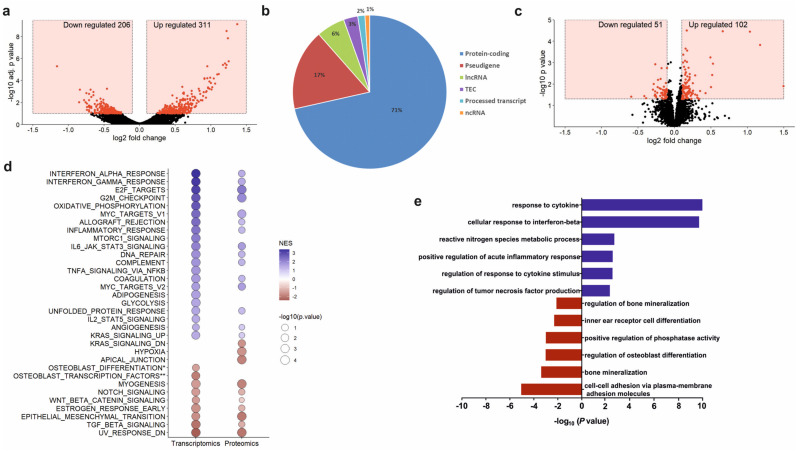
RM1-BM EVs affect the osteoprogenitor transcriptome and proteome. (**a**) Determination of differentially expressed genes (DEGs) represented as volcano plot of log_2_ fold change (x-axis) and statistical significance (y-axis) of PCa-EV treated vs. untreated osteoprogenitors. Red = DEGs. (**b**) Pie chart of the distribution (expressed in percentage) of detected genes into six summarized transcript types: protein-coding, pseudogenes, long-non coding RNA (lncRNA), Tec family tyrosine kinases (TEC), processed transcript, and non-coding RNA (ncRNA). (**c**) Determination of DAPs represented as volcano plot of log_2_ fold change (x-axis) and statistical significance (y-axis) of PCa-EV treated vs. untreated osteoprogenitors. Red = DAPs. (**d**) GSEA of transcriptomic and proteomic enriched pathway. NES: normalized enrichment score; *p*-value (−log10). (**e**) Simplified graphical representation of overrepresented GO terms that are upregulated (blue) and under-regulated (red) in EV-treated osteoprogenitors vs. untreated controls. GO categories for each function were sorted based on the GO enrichment test *p*-value (−log10).

To assess whether the alteration of osteoprogenitor activity after EV exposure was mirrored at the transcriptome and proteome level, we performed a genome-wide RNA sequencing as well as a proteome analysis of seven days differentiated osteoprogenitors treated with PCa-EVs. Of the 20,750 genes profiled in next generation sequencing a total of 517 differentially expressed genes (DEGs) were identified, with 311 being upregulated and 206 being down-regulated (Figure 4a). Among all the DEGs, 71% encode for proteins, 17% for pseudogenes, and 6% for long-non coding RNA. Minor fractions encode for Tec family tyrosine kinases (3%), processed transcripts (2%), and other non-coding RNAs (1%) (Figure 4b, Table S2). Proteomic analysis identified 3604 proteins, of which 3438 could be mapped to the transcriptomics data (Table S3). Proteomic analyses revealed changes in the abundance of 153 mapped proteins after PCa-EV exposure, of which 102 were up-regulated and 51 down-regulated in abundance (Figure 4c). In addition, to investigate molecular alterations in the biological processes of osteoblasts after RM1-BM-EVs uptake, we performed gene set enrichment analysis (GSEA) of whole expression patterns of transcriptomics and proteomics analyses (Table 1 and Table S4). Results revealed good accordance of results of both omics technologies and an over-representation of genes and proteins involved in the inflammatory response, including interferon-alpha and -gamma (IFN-α, IFN-γ) response, tumor necrosis factor-alpha (TNF-α) signaling, as well as interleukin (IL)-6 and Janus kinase (JAK) signal transducer and activator of transcription 3 (STAT3) pathway. Moreover, GSEA confirmed significant downregulation of genes and proteins involved in late osteoblastic differentiation, Wnt signaling, and transforming growth factor-beta (TGF-β) signaling (Figure 4d).

These data were consistent with the gene ontology (GO) analysis (complete list in Table S5) which demonstrated that genes upregulated in EV-treated osteoprogenitors were involved in positive regulation of acute inflammatory response and cytokines stimuli response. By contrast, genes involved in bone mineralization (e.g., *Bmp2* and Krüppel-like factor (*klf)* 10), positive regulation of phosphatase activity, and osteoblastic differentiation were downregulated in EV-treated osteoblast precursors (Figure 4e). Thus, transcriptomic profiling, as well as proteomics, reflect the inhibitory function of PCa-EVs on osteoprogenitors and suggests that EVs trigger a proinflammatory phenotype in osteoblastic cells, potentially supporting tumor cell progression and homing to the bone.

### 3.6. Cancer-Secreted miRNAs Transferred via EVs Impair Osteogenesis and Osteoblast Differentiation

**Figure 5:**
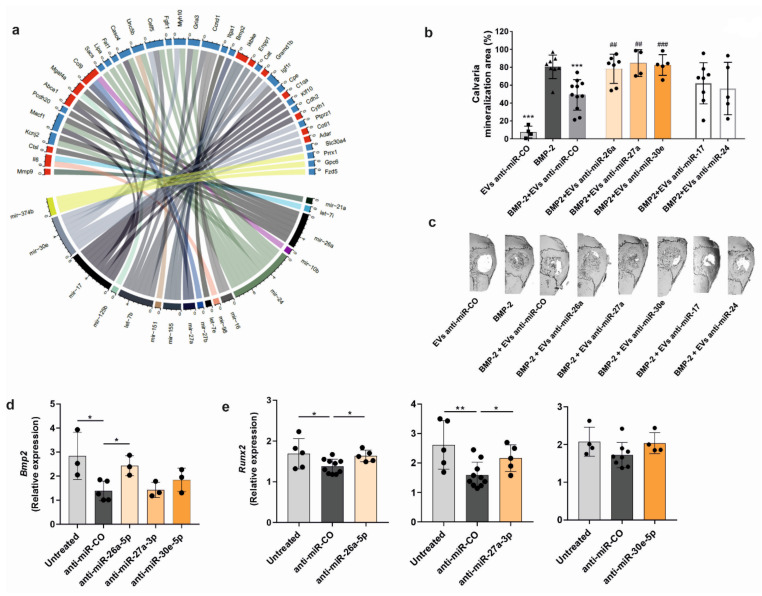
RM1-BM-derived miRNAs transferred via EVs impair bone formation and osteogenic differentiation. (**a**) Chord diagram showing the relationship between PCa-EVs miRNAs and osteoblast target genes. Larger segments indicate that one miRNA has many target genes (lower side) or that a gene is predicted to be a target of many miRNAs (upper side). Upregulated genes of EV-treated osteoblast precursors are colored in red, while the down-regulated genes are colored in blue. To create the plot, we considered only miRNAs with over 2000 reads per million (50 miRNAs) and osteoblastic genes with adjusted *p*-value < 0.05. (**b**) Quantitative analyses of newly-formed bone at the defect sites after 21 days of sponge implantation. Sponges were loaded with BMP-2, or EVs derived from PCa cells which were previously transfected with non-targeting anti-miR control (anti-miR-CO) or targeting anti-miRNA for miR-17, miR-24, miR-26a, miR-27a, and miR30e (*n* = 4–11). Squares: RM1-BM EVs with siCo, triangle: BMP-2, circles: BMP-2 with RM1-BM EVs with respective siRNAs. (**c**) 3D micro-computed tomography images of bone formation at the critical-sized calvarial defects. (**d**,**e**) Expression of the osteoblastic markers *Bmp2* and *Runx2* in osteoprogenitors treated with RM1-BM-EVs with anti-miR-CO or EVs deficient of miR-26a-5p, miR-27a-3p, or miR-30e-5p vs. untreated cells (*n* = 3–10). Circles indicate individual data points. Statistical analysis was performed by one-way ANOVA with Tukey’s post hoc test (**b**) or unpaired Student’s *t*-test (**d**,**e**). Data are presented as mean ± SD. Statistical significance is denoted: *** *p* < 0.001 vs. BMP-2 treated group and ## *p* < 0.01; ### *p* < 0.001 vs. BMP-2+ EVs anti-miR-CO group (**b**). * *p* < 0.05; ** *p* < 0.01 vs. controls (**d**,**e**).

To obtain deeper insights into how PCa-EVs specifically promote the described functional changes in osteoprogenitors, we focused on miRNAs, as exosomal miRNAs have been identified as critical mediators in cell-cell cross-talk [39]. Therefore, we examined the expression profile of miRNAs in RM1-BM EVs by performing small RNA-sequence analysis of the isolated vesicles. Sequencing showed an abundance of over 600 miRNAs selectively packaged into RM1-BM EVs (Table S6). To identify which of the miRNAs could account for the gene expression changes in EV-treated osteoprogenitors, we selected the top 50 most highly expressed exosomal miRNAs (RPM > 2000), identified their known targets, and integrated that information with the differential changes in the signature of EV-treated osteoprogenitors (Figure 5a). Because miRNA regulation of targets can be redundant, only osteoblastic genes targeted by multiple miRNAs were considered. This analysis highlighted seventeen miRNAs, which could explain the gene regulation identified in EV-treated osteoprogenitors. Five of these exosomal miRNAs—miR-17-5p, miR-24-3p, miR-26a-5p, miR-27a-3p, and miR-30e-5p—were then selected for further investigation based on published association with osteogenesis and the number of genes targeted in osteoblasts.

To examine their impact on bone mineralization, the expression of the five candidate miRNAs was abrogated in RM1-BM cells using miRNA inhibitors. EVs were then collected, and successful knockdown was confirmed by qPCR (Figure S1). EVs deficient in either one of the five miRNAs were used in the calvarial defect model. While EVs with the anti-miR control inhibited BMP-2-induced bone formation by 39%, EVs with the inhibition of miR-26a-5p, miR-27a-3p, and miR-30e-5p were not able to suppress bone formation (Figure 5b,c). By contrast, knockdown of miR-17-5p and miR-24-3p in EVs showed a similar suppression of bone formation as EVs with the anti-miR control (Figure 5b,c). In line with these data, osteoprogenitors treated with PCa-EVs in vitro showed low expression of *Bmp2* and *Runx2,* two genes that are the main targets of miR-30e-5p, miR-26a-5p, and miR-27a-3p (Figure 5d,e). Knockdown of exosomal miR-26a-5p rescued gene expression of *Bmp2* in EV-treated osteoprogenitors, while miR-30e-5p and miR-27a-3p inhibition showed only a tendency that did not reach statistical significance (Figure 5d). Besides, knockdown of both miR-26a-5p and miR-27a-3p, but not miR-30e-5p, was able to restore the expression of *Runx2* in osteoprogenitors (Figure 5e). Overall, these data indicate that PCa-EVs modulate osteoblast behavior by releasing miRNAs, resulting in a significant decrease in osteogenic gene expression and bone formation.

### 3.7. PCa-Derived EVs Educate Osteoprogenitors to Support Tumor Activity In Vitro

**Figure 6:**
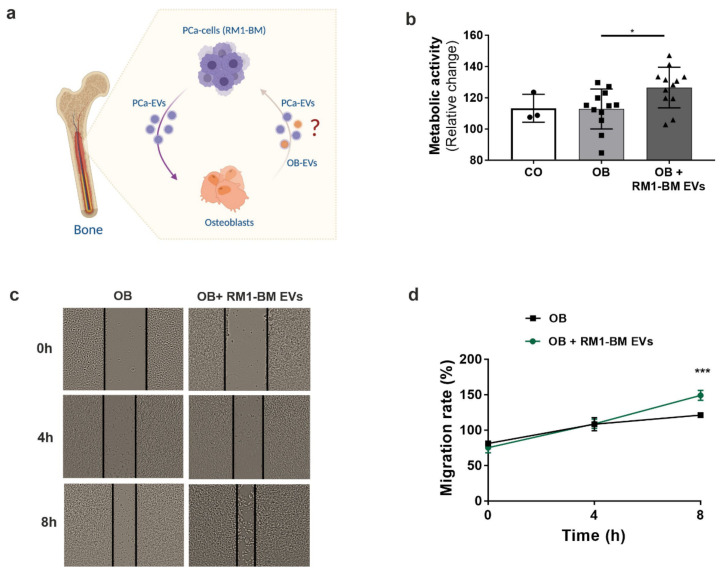
Education of osteoprogenitors via RM1-BM-EVs promotes tumor cell metabolic activity and migration in vitro. (**a**) Schematic representation of the vicious cycle between osteoprogenitors and PCa cells mediated by EVs (image was created with BioRender.com). (**b**) RM1-BM metabolic activity was evaluated based on CellTiter Blue assay comparing untreated tumor cells (CO), cells treated with supernatant from unconditioned osteoprogenitors (OB), or cells treated with supernatant from EVs-conditioned osteoprogenitors (OB + RM1-BM EVs) (*n* = 3–12). Circles: control, squares: osteoblast supernatant, triangle: osteoblast supernatant with RM1-BM EVs. (**c**) Representative bright-field images (20×) of the scratch assay of RM1-BM cells treated with supernatant from unconditioned osteoprogenitors or EVs-conditioned osteoprogenitors after 0, 4, and 8 h. (**d**) Quantification of migration of RM1-BM cells indicated as percentages of scratch closure and calculated as follows: % of scratch closure = x−y/x, where (x) is a distance between edges of the wound, and (y) is the distance which remained cell-free during cell migration. Results are representative of more than three independently executed experiments. Statistical analysis was performed by one-way ANOVA with Tukey’s post hoc test (**b**) or unpaired Student’s *t*-test (**d**). Data are presented as mean ± SD. Statistical significance is denoted: * *p* < 0.05; *** *p* < 0.001vs RM1-BM cells treated with EVs-free osteoblast supernatant.

Finally, as tumor-derived EVs are crucial for creating a conducive milieu for tumor colonization, we next asked whether the molecular changes in EV-treated osteoprogenitors contribute to a supportive growth environment for the tumor cells (Figure 6a). Interestingly, treatment of RM1-BM cells with EV-educated osteoprogenitor supernatant induced a significant increase in the metabolic activity after 24 h of exposure compared to RM1-BM cells treated with osteoprogenitors supernatant without EV pre-conditioning (Figure 6b). Consistent with these results, migration of tumor cells was 20% higher when treated with supernatant from EV-educated osteoprogenitors for 8 h as compared to the control (Figure 6c,d), suggesting that PCa-EVs might promote tumor aggressiveness and metastasis through educating osteoblasts.

## 4. Discussion

Skeletal metastases represent one of the most severe complications in PCa, with an incidence of up to 90% in patients at a late stage of the disease [2]. Within the bone metastatic niche, bidirectional interactions between tumor cells and osteoblastic cells actively contribute to PCa osteotropism [40]. Recently, PCa-EVs have been recognized as crucial signaling mediators in cell-cell communication and to modify the bone microenvironment by creating conditions conducive to tumor growth and development [8]. However, how PCa-derived EVs influence the metastatic bone phenotype is not fully understood. To address this question, we investigated the potential contribution of PCa-EVs in transferring molecular information to osteoblastic cells and elucidated how such dynamic interplay affects the physiological behavior of the recipient cells. Consistent with previous studies [15], we showed that PCa-EVs spontaneously crossed cellular membranes in osteoprogenitors, which resulted in a functional delivery of their cargo. Nevertheless, internalization of RM1-BM EVs was associated with a more than two-fold increase in osteoprogenitor proliferation, enhanced cell vitality and metabolic activity, and a reduced mineralization capacity, suggesting the ability of EVs to manipulate bone-forming cells and boost the vicious cycle of skeletal metastases. 

In humans, dissemination of PCa cells to bone frequently leads to pathological alteration in osteoblast function [41]. Consistent with this clinical evidence, we found that EVs derived from RM1-BM cells have the biological potential to promote changes in the genetic signature of the osteoblast precursors. Indeed, EV internalization lowered the transcript abundance of osteogenic markers and pathways (e.g., Wnt/β-catenin and TGF-β/BMP), thereby inhibiting osteoblast maturation and in vitro mineralization capacity. Consistently, in vivo analysis of bone formation in two complementary mouse models that assess osteoblast performance in vivo showed impairment in osteoblast function, reflected by a significant reduction of ectopic and BMP-2-induced bone formation. Thus, these inhibitory effects emphasize the importance of cancer EVs in cellular cross-talk and advocate their impact on the physiological behavior of distant target cells.

Interestingly, these data are in contrast with prior studies, in which vesicles released by human PCa cell line MDA-PCa-2b and C4-2B exhibit osteoblastic phenotype, by promoting osteogenesis differentiation in human osteoprogenitors cell lines [18,42]. Nevertheless, EVs derived from human PC3 cells have been reported to either inhibit or promote osteoblastogenesis when applied to murine osteoblast cell lines [43,44].

These discrepancies might be explained by the use of different cellular subclones and by the combination of cell lines with a different species of origin (human and murine), while in our study, we used a murine-only system. Notably, the choice of the cell type is of paramount importance for obtaining specific EV-mediated responses on recipient cells. Indeed, the functional heterogeneity of EV-cargo is tightly correlated to the tissue and species of origin and might explain the ambiguous outcomes that several publications have reported until now.

Over the past years, several reports have demonstrated the ability of cancer-EVs to reprogram cells toward a pro-tumorigenic phenotype, thereby endowing the tumor with a survival advantage over other competing cells within a foreign microenvironment [45]. For example, EVs have been reported to activate inflammatory responses and modulate the innate and adaptive immune system [46,47]. Thus, overexpression of inflammation-related genes observed in osteoprogenitors after EVs uptake may be interpreted as a strategy implemented by PCa cells to escape immune destruction. Notably, accumulation of inflammatory mediators, such as transcription factors NFκB and STAT3 as well as cytokines IL-6 and TNF-α, has been linked to local and systemic immunosuppression associated with the progression of cancer [48]. Furthermore, sustained exposure to inflammatory stimuli contributes to tumor infiltration and colonization of the bone [49], a phenomenon that might explain the increased expression of genes involved in angiogenesis. In addition to the transcriptomic changes in EV-treated osteoprogenitors that may argue for a tumor-promoting phenotype, our in vitro analysis provides a proof-of-principle that the education of osteoprogenitors with PCa-derived EVs enhances tumor cell metabolic activity and supports migration of cancer cells. Certainly, further investigations are needed to validate whether these results are reproducible in an in vivo setting of osteotropism and colonization of bone and to decipher the molecular mechanisms underlying the tumor-promoting effects of PCa-EV educated osteoblastic cells.

Among all the molecules carried by tumorigenic EVs, RNA sequencing and profiling studies have shown an abundance of miRNAs [50,51] that are involved in tumor development, progression, and metastasis [19,52]. However, the role of cancer-secreted miRNAs in the bone metastatic phenotype is not completely understood. In our study, we identified three distinct miRNAs, miR-26a-5p, miR-27a-3p, and miR-30e-5p that are highly expressed in the RM1-BM EVs and involved in the suppression of osteoblastic function. Previous studies have already demonstrated that miR-27a-3p negatively regulates differentiation of osteoprogenitors by controlling osterix (Osx) expression, an early response gene essential for bone formation [53]. In contrast, miR-26a-5p, as well as miR-30e-5p, have been shown to support osteogenic differentiation of both bone marrow-derived mesenchymal stromal cells and adipocytes [54,55]. However, their role in the context of the PCa remains controversial [56,57]. Within our study, knockdown of miR-26a-5p, miR-27a-3p, and miR-30e-5p each reversed the negative effect on bone repair that EVs have shown when combined with BMP-2. Consistently, in vitro analysis revealed that the inhibition of exosomal miR-26a-5p, but not miR-27a-3p or miR-30e-5p, rescued the expression of *Bmp2* in osteoprogenitors while both miR-26a-5p and miR27a-3p were able to restore the expression of the osteogenic marker *Runx2*. Altogether, these findings highlight three miRNAs that are important for osteoblast function and underline the notion that bone remodeling in osteoblastic bone lesions can be regulated by PCa-EV-derived miRNAs, whose major function is to convey phenotypic transforming signals to non-malignant cells.

Besides the strengths that include detailed investigations of the PCa-EVs cargo, the comprehensive characterization of osteoblast interactions, and the use of a syngeneic murine model, our study has potential limitations. First, the effect of EVs on osteoblast precursors was evaluated using a single murine osteotropic cancer cell line. This cell line was selected because it can be used in syngeneic mouse models with an intact immune system. It would be informative to investigate the biological properties of EVs derived from non-osteotropic tumors; however, non-osteotropic tumor cell lines from C57BL/6J mice are scarce. Our study also lacks healthy controls, mainly due to difficulties in finding murine non-cancerous murine prostate epithelial cells compared to human cell lines. Finally, comprehensive analyses are still necessary to accelerate the translation of EV-based biomarkers from the bench to the bedside. Indeed, since all types of cells release vesicles, the EVs derived from body liquids are more heterogeneous than those isolated in cell culture, leading to a possible discrepancy between results.

Collectively, our findings suggest that PCa-EVs inhibit osteoblastogenesis and impair bone mineralization by transferring miRNAs, thereby potentially contributing to the formation of PCa-bone lesions. A better understanding of these processes and associated immune response may result in novel targeted approaches against bone metastases in PCa.

## Figures and Tables

**Table 1:** Summary of the results obtained from gene set enrichment analysis.

HALLMARK Pathway	SIZE	NES	*p*-Value	FDR *q*-Value
**Down-regulated**				
UV_RESPONSE_DN	140	−2.43	0.001	0.001
TGF_BETA_SIGNALING	54	−2.11	0.001	0.001
OSTEOBLAST_TRANSCRIPTION_FACTORS **	14	−1.96	0.002	0.001
EPITHELIAL_MESENCHYMAL_TRANSITION	184	−1.71	0.002	0.006
ESTROGEN_RESPONSE_EARLY	174	−1.69	0.001	0.0062
WNT_BETA_CATENIN_SIGNALING	39	−1.54	0.021	0.020
NOTCH_SIGNALING	32	−1.54	0.002	0.017
MYOGENESIS	171	−1.53	0.001	0.0167
OSTEOBLAST_DIFFERENTIATION *	12	−1.47	0.059	0.029
**Upregulated**				
INTERFERON_ALPHA_RESPONSE	89	3.40	0.001	0.001
INTERFERON_GAMMA_RESPONSE	185	3.28	0.001	0.001
E2F_TARGETS	194	3.15	0.001	0.001
G2M_CHECKPOINT	190	2.81	0.001	0.001
OXIDATIVE_PHOSPHORYLATION	179	2.78	0.001	0.001
MYC_TARGETS_V1	183	2.49	0.001	0.001
ALLOGRAFT_REJECTION	157	2.47	0.001	0.001
INFLAMMATORY_RESPONSE	177	2.33	0.001	0.001
MTORC1_SIGNALING	193	2.21	0.001	0.001
IL6_JAK_STAT3_SIGNALING	78	2.11	0.001	0.001
DNA_REPAIR	138	1.90	0.001	0.001
COMPLEMENT	164	1.84	0.001	0.001
TNFA_SIGNALING_VIA_NFKB	193	1.80	0.001	0.001
COAGULATION	100	1.75	0.001	0.003
MYC_TARGETS_V2	56	1.70	0.002	0.004
ADIPOGENESIS	184	1.63	0.001	0.008
GLYCOLYSIS	183	1.55	0.001	0.017
PROTEIN_RESPONSE	101	1.53	0.002	0.019
IL2_STAT5_SIGNALING	184	1.47	0.007	0.031
ANGIOGENESIS	31	1.44	0.061	0.037
KRAS_SIGNALING_UP	172	1.44	0.002	0.037

*p*-value: corrected with Bonferroni post hoc test. NES: normalized enrichment score; FDR: false discovery rate. * Dataset from Park et al.; ** Dataset from Zaidi et al.

## Data Availability

Data supporting reported results will be provided by the authors upon request.

## Supplementary Materials

The following supporting information can be downloaded at: https://www.mdpi.com/article/10.3390/ijms23031285/s1.
