# Supplementary material for: Exosomal miRNAs from Prostate Cancer Impair Osteoblast Function in Mice
Source: Int J Mol Sci. 2022 Jan 24;23(3):1285. doi: 10.3390/ijms23031285 (PMC8836054; doi:10.3390/ijms23031285)
Supplement: Supplementary file 1 [file ijms-23-01285-s001.zip › Supplementary Figure S1.pdf]

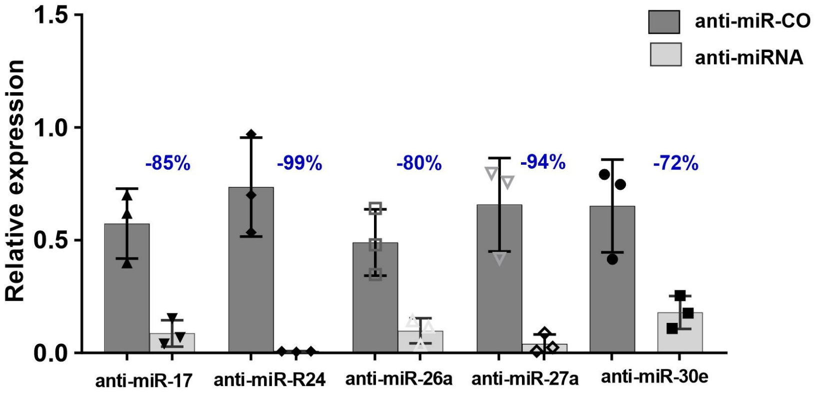

Supplementary Figure S1. Knockdown efficiency of anti-miRNA targeting EV-derived miRNAs from RM1-BM. RM1-BM were transfected with non-targeting anti-miR-control or targeting miRNA inhibitors for miR-17-5p, miR-24-3p, miR-26a-5p, miR-27a-3p, miR-30e-5p. After 48 h, mRNA was isolated, and qPCR analyses were performed. Data are presented as mean ± SD (n=3). The percentage of knockdown was calculated for the cells treated with targeting vs. non-targeting anti-miR CO for each exosomal miRNAs.
