# Supplementary material for: Exosomal miRNAs from Prostate Cancer Impair Osteoblast Function in Mice
Source: Int J Mol Sci. 2022 Jan 24;23(3):1285. doi: 10.3390/ijms23031285 (PMC8836054; doi:10.3390/ijms23031285)
Supplement: Supplementary file 1 [file ijms-23-01285-s001.zip › Supplementary Table S2.pdf]

Suppl. Table S2. Differentially expressed genes in osteoprogenitors treated with RM1-BM EVs.

Gene_Symbol	log2FoldChange	padj
Pcdh20	-1.16	4.99E-06
Sema3d	-0.85	0.009494
Ptprz1	-0.84	0.000685
Igfbp2	-0.78	0.006152
Atoh8	-0.76	0.009491
Thsd4	-0.75	0.001378
Celf5	-0.74	0.012577
Slc13a5	-0.74	0.025799
Nell2	-0.74	0.045318
Igsf9b	-0.72	0.023355
Clec11a	-0.71	0.001588
Npnt	-0.71	0.006152
Lmo7	-0.71	0.018204
Shank1	-0.71	0.019295
Tnfrsf19	-0.71	0.024288
Col25a1	-0.71	0.048557
Ptprv	-0.7	0.021159
Bmp2	-0.7	0.02901
Fam129a	-0.69	0.000685
Epha3	-0.68	0.003084
Col8a2	-0.68	0.023866
Cobll1	-0.68	0.031104
Irx3	-0.65	0.001105
Fat1	-0.65	0.002112
Fzd5	-0.63	0.013333
Ano3	-0.63	0.045713

Sacs	-0.61	0.020581
Fat4	-0.61	0.024161
Cat	-0.6	0.006504
Igsf10	-0.59	0.015244
Rap1gap	-0.59	0.038072
Myom1	-0.59	0.04006
Slit3	-0.56	0.012966
Cdh2	-0.56	0.027717
Cdh11	-0.55	0.004429
Ddr2	-0.55	0.019212
Itga1	-0.54	0.00823
Ahnak	-0.54	0.012966
Dpysl3	-0.54	0.021159
Ptpn13	-0.54	0.037352
Tiam2	-0.54	0.041026
Setbp1	-0.54	0.046022
Adgrf5	-0.53	0.025356
Myo6	-0.53	0.044729
Gria3	-0.52	0.020581
Ltbp1	-0.52	0.023526
Casc4	-0.52	0.033809
Gpc6	-0.52	0.039801
Id3	-0.51	0.005533
Gclm	-0.51	0.009655
Sytl2	-0.51	0.035367
Cspg4	-0.51	0.049422
Hspg2	-0.5	0.012966
Kcnj2	-0.49	0.028001
Dnm3os	-0.49	0.034137
Scarf2	-0.49	0.044729
Rnd3	-0.49	0.045811

Ptpn14	-0.48	0.019375
Qsox1	-0.48	0.029135
Unc5b	-0.47	0.013707
Prrx1	-0.47	0.024967
Klf10	-0.47	0.042421
Postn	-0.46	0.000542
Fgfr1	-0.45	0.016333
Slc30a4	-0.45	0.023142
Ank	-0.45	0.036827
Cacna1g	-0.45	0.041419
Utrn	-0.45	0.045767
Dkk3	-0.45	0.045811
Akap12	-0.44	0.022634
Myh10	-0.44	0.036884
Ccnd2	-0.42	0.037181
Kirrel	-0.42	0.038614
Rai14	-0.42	0.046022
Igf1r	-0.42	0.046462
Macf1	-0.41	0.028827
Cmtm4	-0.41	0.039029
Ccnd1	-0.41	0.048307
Msrb3	-0.4	0.02507
Neo1	-0.4	0.038614
Lipa	-0.4	0.039801
Dst	-0.39	0.043191
Cpe	-0.38	0.031256
Thbs2	-0.38	0.049422
Pkd1	-0.37	0.041328
Golim4	-0.36	0.031859
Cdc42bpa	-0.36	0.043191
Pltp	0.32	0.04184

Rps3	0.33	0.04028
Cyth1	0.34	0.049398
Ncapg2	0.35	0.031104
Atp13a2	0.36	0.016844
Rps14	0.36	0.022582
Abca1	0.36	0.023526
Fxyd5	0.36	0.048412
Pld4	0.37	0.021159
Gm6204	0.37	0.024572
Tmsb4x	0.37	0.046022
C1qb	0.38	0.020581
Rpl41	0.38	0.030892
Cxcr4	0.39	0.020581
Ctsl	0.4	0.035113
C1qc	0.42	0.024155
Cd72	0.42	0.03273
Ctsc	0.42	0.03883
Cyba	0.42	0.048943
Ifi207	0.43	0.018204
Trafd1	0.43	0.023526
Aoah	0.43	0.049234
Cotl1	0.44	0.028827
Ly86	0.45	0.028827
Cd86	0.45	0.03883
Cfp	0.46	0.002256
Ikbke	0.46	0.012966
Apobec3	0.46	0.034733
Enpp1	0.46	0.03883
Ogfr	0.46	0.045713
Mgat4a	0.48	0.021159
Lgmn	0.48	0.022582

Irf7	0.48	0.039345
Hexb	0.48	0.042421
Irf9	0.49	0.026945
Fbln2	0.49	0.028827
Il4ra	0.5	0.025289
Rnf114	0.5	0.025799
Gramd1b	0.51	0.025289
Psme1	0.51	0.044729
C1qa	0.52	0.003924
Tmem86a	0.52	0.00823
Adar	0.52	0.025955
Eif2ak2	0.55	0.012884
Ly6a	0.55	0.016732
Tspo	0.55	0.022529
Zbp1	0.55	0.037352
Abi3	0.56	0.013378
Ms4a6c	0.56	0.014545
Tgm2	0.56	0.021159
Cdc25b	0.56	0.022382
Ccl9	0.57	0.005882
Pilra	0.57	0.006152
Sgk1	0.57	0.009144
1600014C10Rik	0.57	0.024288
Pla1a	0.57	0.024793
Gngt2	0.57	0.026746
Tpx2	0.57	0.033494
Ifi213	0.58	0.031859
Aif1	0.59	0.005705
Trpm2	0.59	0.014545
Ms4a6d	0.59	0.01614
Oasl2	0.59	0.021294

Iqgap3	0.59	0.031859
Ifit3b	0.59	0.045507
Ms4a4a	0.6	0.000367
Nlrc5	0.6	0.038072
Acod1	0.6	0.03883
Foxm1	0.6	0.04184
Il21r	0.61	0.001452
Dtx3l	0.61	0.004354
Selplg	0.61	0.004354
Ccr5	0.61	0.006196
Cenpe	0.61	0.034365
Ms4a6b	0.62	0.001244
H2-K1	0.62	0.001588
B2m	0.62	0.004875
Nrep	0.62	0.028001
Pnp	0.63	0.016589
Ifi44	0.63	0.023526
Slfn5	0.63	0.031859
Prdx5	0.64	0.005053
Psmb10	0.64	0.006032
Sp110	0.64	0.024793
Parp10	0.65	0.012577
Ncapg	0.65	0.02991
Bub1	0.65	0.03883
Ccr1	0.65	0.04594
Kntc1	0.66	0.026436
Top2a	0.67	0.002415
Sirpb1c	0.67	0.004664
Kif11	0.67	0.013342
Tmem221	0.67	0.04184
Pi15	0.68	0.00918

Psmb9	0.68	0.012878
AW112010	0.68	0.039703
Fanci	0.68	0.045811
Ccna2	0.69	0.014545
Epsti1	0.69	0.020581
Nuf2	0.69	0.02507
Igtp	0.69	0.025289
H2-Q6	0.7	0.02991
Irgm2	0.71	0.004429
Trim34a	0.71	0.006152
Ifit1	0.71	0.013342
Plk1	0.72	0.011602
Acp5	0.72	0.038614
Uba7	0.73	0.000857
Parp9	0.73	0.001591
Pf4	0.73	0.014208
Ifi27l2a	0.73	0.017366
Cep55	0.73	0.030892
H2-Q4	0.74	0.00287
Ly6e	0.74	0.012884
Medag	0.75	0.006089
Clu	0.75	0.006089
Il6	0.76	0.006152
Slfn8	0.77	0.002956
Kif2c	0.77	0.024967
Gbp2	0.78	0.003924
Plxna4os1	0.78	0.024288
BC147527	0.78	0.024288
Mcm10	0.79	0.019066
Kif18b	0.79	0.020581
Ifi205	0.8	0.011648

Gbp3	0.81	0.006196
Mndal	0.83	0.000542
Oas1b	0.83	0.008176
Slfn9	0.84	0.000857
Herc6	0.85	0.001588
Cxcl3	0.86	0.006032
Gm1966	0.88	0.006152
Cxcl10	0.88	0.006362
Rtp4	0.89	0.002549
Chil1	0.9	0.001109
Folr2	0.91	6.04E-05
Ifi203	0.91	6.86E-05
Bach2os	0.93	0.001105
Mx2	0.93	0.003561
Ccl5	0.94	0.000944
Gbp7	0.95	4.99E-06
Ctla2a	0.95	6.17E-05
Fcgr4	0.96	0.000424
Saa3	0.96	0.000496
Gbp5	0.98	0.000124
Lcn2	0.98	0.000542
Gm5431	0.99	5.36E-05
Cd300lf	1	0.000167
Cdh5	1.01	0.000648
Pecam1	1.02	0.000396
Mmp9	1.04	6.5E-05
Treml2	1.06	0.000185
Trim30c	1.09	2.74E-05
Ly6i	1.11	2.56E-05
Gm12250	1.17	3.84E-06
Gm4951	1.2	2.72E-06

Cd38	1.21	6.9E-06
Oas2	1.22	3.08E-09
Phf11a	1.22	3.7E-06
Gm21188	1.24	1.41E-08
Pydc3	1.25	1.74E-06
Gm36161	1.37	7.46E-10
