# Supplementary material for: Exosomal miRNAs from Prostate Cancer Impair Osteoblast Function in Mice
Source: Int J Mol Sci. 2022 Jan 24;23(3):1285. doi: 10.3390/ijms23031285 (PMC8836054; doi:10.3390/ijms23031285)
Supplement: Supplementary file 1 [file ijms-23-01285-s001.zip › Supplementary Table S4.pdf]

Suppl. Table S4. GSEA of whole expression patterns of transcriptomic and proteomic analyses.

	Transcriptomics				*Proteomics*			
**HALLMARK**	**SIZE**	**NES**	**p-value**	**FDR q-value**	**SIZE**	**NES**	**p-value**	**FDR q-value**
UV_RESPONSE_DN	140	-2.43	0.001	0.001	33	-2.06	0.001	0.001
TGF_BETA_SIGNALING	54	-2.11	0.001	0.001	13	-1.24	0.232	0.390
EPITHELIAL_MESENCHYMAL_TRANSITION	184	-1.71	0.002	0.006	80	-2.04	0.001	0.001
ESTROGEN_RESPONSE_EARLY	174	-1.69	0.001	0.0062	28	-1.17	0.245	0.437
WNT_BETA_CATENIN_SIGNALING	39	-1.54	0.021	0.02	8	-0.75	0.765	0.984
NOTCH_SIGNALING	32	-1.54	0.002	0.017	5	-1.2	0.262	0.427
MYOGENESIS	171	-1.53	0.001	0.0167	57	-1.88	0.001	0.003
OSTEOBLAST_TRANSCRIPTION_FACTORS**	14	-1.96	0.002	0.001				
OSTEOBLAST_DIFFERENTIATION*	12	-1.47	0.059	0.029				
APICAL_JUNCTION					67	-1.89	0.001	0.004
HYPOXIA					63	-1.66	0.001	0.049
KRAS_SIGNALING_DN					13	-1.66	0.020	0.041
INTERFERON_ALPHA_RESPONSE	89	3.4	0.001	0.001	30	1.42	0.086	0.194
INTERFERON_GAMMA_RESPONSE	185	3.28	0.001	0.001	61	1.46	0.041	0.174
E2F_TARGETS	194	3.15	0.001	0.001	80	2.24	0.001	0.001
G2M_CHECKPOINT	190	2.81	0.001	0.001	72	1.98	0.001	0.002

OXIDATIVE_PHOSPHORYLATION	179	2.78	0.001	0.001				
MYC_TARGETS_V1	183	2.49	0.001	0.001	172	1.54	0.001	0.120
ALLOGRAFT_REJECTION	157	2.47	0.001	0.001	59	1.18	0.205	0.307
INFLAMMATORY_RESPONSE	177	2.33	0.001	0.001	31	1.24	0.190	0.288
MTORC1_SIGNALING	193	2.21	0.001	0.001				
IL6_JAK_STAT3_SIGNALING	78	2.11	0.001	0.001	17	1.7	0.017	0.054
DNA_REPAIR	138	1.9	0.001	0.001	58	1.32	0.108	0.278
COMPLEMENT	164	1.84	0.001	0.001	70	1.31	0.092	0.258
TNFA_SIGNALING_VIA_NFKB	193	1.8	0.001	0.001				
COAGULATION	100	1.75	0.001	0.003	38	1.26	0.157	0.302
MYC_TARGETS_V2	56	1.7	0.002	0.004	28	1.66	0.026	0.061
ADIPOGENESIS	184	1.63	0.001	0.008				
GLYCOLYSIS	183	1.55	0.001	0.017				
UNFOLDED_PROTEIN_RESPONSE	101	1.53	0.002	0.019	62	1.18	0.203	0.326
IL2_STAT5_SIGNALING	184	1.47	0.007	0.031				
ANGIOGENESIS	31	1.44	0.061	0.037	14	0.91	0.573	0.771
KRAS_SIGNALING_UP	172	1.44	0.002	0.037	61	0.89	0.592	0.990

P value: corrected with Bonferroni post hoc test. NES: Normalized enrichment score; FDR: false discovery rate.
