# Supplementary material for: Exosomal miRNAs from Prostate Cancer Impair Osteoblast Function in Mice
Source: Int J Mol Sci. 2022 Jan 24;23(3):1285. doi: 10.3390/ijms23031285 (PMC8836054; doi:10.3390/ijms23031285)
Supplement: Supplementary file 1 [file ijms-23-01285-s001.zip › Supplementary Table S5.pdf]

Suppl. Table S5. Summary of the results obtained from gene ontology analysis.

(a) Up-regulated genes in RM1-BM EVs-treated osteoprogenitors.

GO	Function	Size	p-value	Genes
BP	Cell-cell adhesion via plasma-membrane adhesion molecules	7	8.8 E-6	[Bmp2. Cdh11. Cdh2. Fat4. Igsf9b. Pcdh20. Scarf2]
BP	Response to cytokine	23	2.4 E-17	[Acp5. Ccl5. Ccr1. Ccr5. Cxcl10. Cxcl3. Gbp2. Gbp3. Gbp5. Gbp7. Gm4951. Ifi203. Ifi205. Ifit1. Igtp. Il21r. Il6. Irgm2. Mx2. Nlrc5. Parp9. Pf4. Saa3]
BP	Cellular response to cytokine stimulus	19	3.4 E-14	[Ccl5. Ccr1. Ccr5. Cxcl10. Cxcl3. Gbp2. Gbp3. Gbp5. Gbp7. Gm4951. Ifi203. Ifi205. Ifit1. Igtp. Il21r. Il6. Nlrc5. Pf4. Saa3]
BP	Cellular response to interferon-beta	7	1.6 E-10	[Gbp2. Gbp3. Gm4951. Ifi203. Ifi205. Ifit1. Igtp]
BP	Defense response to protozoan	5	1.2 E-6	[Gbp2. Gbp3. Gbp7. Il6. Irgm2]
BP	Sister chromatid segregation	7	6.7 E-6	[Bub1. Cenpe. Kif18b. Kif2c. Ncapg. Plk1. Top2a]
BP	Regulation of viral genome replication	5	9.9 E-5	[Ccl5. Mx2. Oas1b. Parp10. Top2a]
BP	Positive regulation of cell killing	4	7.2 E-4	[B2m. Ccr5. H2-K1. Pnp]
BP	Mitotic cell cycle checkpoint	5	7.3 E-4	[Bub1. Cenpe. Kntc1. Plk1. Top2a]
BP	Chromosome separation	4	1.3 E-3	[Bub1. Cenpe. Plk1. Top2a]
BP	Regulation of chromosome segregation	4	1.6 E-3	[Bub1. Cenpe. Kif2c. Plk1]
BP	Spindle assembly checkpoint	3	1.6 E-3	[Bub1. Cenpe. Plk1]

BP	Mitotic spindle checkpoint	3	1.8 E-3	[Bub1. Cenpe. Plk1]
BP	Reactive nitrogen species metabolic process	4	1.9 E-3	[Acp5. Clu. Il6. Prdx5]
BP	Negative regulation of protein ubiquitination	4	2.0 E-3	[Cenpe. Gbp3. Parp10. Plk1]
BP	Positive regulation of acute inflammatory response	3	2.6 E-3	[Ccl5. Ccr5. Il6]
BP	Regulation of response to cytokine stimulus	3	2.7 E-3	[Ccl5. Nlrc5. Parp9]
BP	Regulation of tumor necrosis factor production	4	4.5 E-3	[Acp5. Ccr5. Clu. Pf4]
BP	Positive regulation of tyrosine phosphorylation of STAT protein	3	4.6 E-3	[Ccl5. Il6. Pecam1]
CC	Symbiont-containing vacuole	4	7.1 E-7	[Gbp2. Gbp3. Gbp7. Irgm2]
CC	MHC class I protein complex	4	8.9 E-6	[B2m. H2-K1. H2-Q4. H2-Q6]
CC	Condensed nuclear chromosome kinetochore	3	7.3 E-5	[Bub1. Cenpe. Plk1]
CC	Kinetochore	6	1.0 E-4	[Bub1. Cenpe. Kif2c. Kntc1. Nuf2. Plk1]
CC	Condensed chromosome outer kinetochore	3	2.8 E-4	[Bub1. Cenpe. Plk1]
CC	Golgi medial cisterna	3	6.4 E-4	[H2-K1. H2-Q4. H2-Q6]
ISP	Response to interferon-gamma	8	1.6 E-9	[Ccl5. Gbp2. Gbp3. Gbp5. Gbp7. Irgm2. Nlrc5. Parp9]
ISP	Positive regulation of leukocyte migration	7	3.9 E-6	[Ccl5. Ccr1. Cxcl10. Cxcl3. Mmp9. Pecam1. Pf4]
ISP	Cellular response to interferon-gamma	5	1.1 E-5	[Gbp2. Gbp3. Gbp5. Gbp7. Nlrc5]

ISP	Positive regulation of leukocyte chemotaxis	5	1.5 E-4	[Ccl5. Ccr1. Cxcl10. Cxcl3. Pf4]
ISP	Positive regulation of monocyte chemotaxis	3	2.8 E-4	[Ccl5. Ccr1. Cxcl10]
ISP	Positive regulation of T cell mediated cytotoxicity	3	9.0 E-4	[B2m. H2-K1. Pnp]
MF	Chemokine activity	4	2.8 E-4	[Ccl5. Cxcl10. Cxcl3. Pf4]
KEGG	TNF signaling pathway	6	8.8 E-5	[Ccl5. Cxcl10. Cxcl3. Gm5431. Il6. Mmp9]

BP = biological processes; CC= cellular components; MF= molecular functions; ISP= immune system process; KEGG= Kyoto Encyclopedia of Genes and Genomes; up-regulated genes cutoff: +0.6 (p-value).

(b) Down-regulated genes in RM1-BM EVs-treated osteoprogenitors.

GO	Function	Size	p-value	Genes
BP	Cell-cell adhesion via plasma-membrane adhesion molecules	7	8.8 E-6	[Bmp2. Cdh11. Cdh2. Fat4. Igsf9b. Pcdh20. Scarf2]
BP	Homophilic cell adhesion via plasma membrane adhesion molecules	5	2.1 E-4	[Cdh11. Cdh2. Fat4. Igsf9b. Pcdh20]
BP	Ureteric bud development	5	2.7 E-4	[Bmp2. Cat. Fat4. Fgfr1. Npnt]
BP	Establishment of nucleus localization	3	2.7 E-4	[Cdc42bpa. Myh10. Slit3]
BP	Bone mineralization	5	4.1 E-4	[Ank. Bmp2. Ddr2. Dnm3os. Klf10]

BP	Regulation of steroid hormone biosynthetic process	3	6.9 E-4	[Bmp2. Dkk3. Igf1r]
BP	Regulation of osteoblast differentiation	5	9.7 E-4	[Bmp2. Ddr2. Fgfr1. Id3. Npnt]
BP	Positive regulation of phosphatase activity	3	1.0 E-3	[Bmp2. Itga1. Npnt]
BP	Positive regulation of cyclin-dependent protein serine/threonine kinase activity	3	1.0 E-3	[Ccnd1. Ccnd2. Pkd1]
BP	Heterophilic cell-cell adhesion via plasma membrane cell adhesion molecules	3	2.4 E-3	[Cdh2. Fat4. Scarf2]
BP	Developmental induction	3	2.8 E-3	[Bmp2. Fgfr1. Fzd5]
BP	Inner ear receptor cell differentiation	3	5.0 E-3	[Fat4. Fgfr1. Myo6]
BP	Branching involved in ureteric bud morphogenesis	3	6.5 E-3	[Bmp2. Fat4. Npnt]
BP	Regulation of bone mineralization	3	7.7 E-3	[Ank. Bmp2. Ddr2]
BP	Positive regulation of osteoblast differentiation	3	8.4 E-3	[Bmp2. Ddr2. Npnt]
CC	Cell-cell contact zone	4	9.7 E-4	[Ahnak. Cdh2. Dst. Kcnj2]
MF	Transmembrane receptor protein tyrosine kinase activity	4	1.0 E-3	[Ddr2. Epha3. Fgfr1. Igf1r]
KEGG	ECM-receptor interaction.	4	2.3 E-3	[Hspg2. Itga1. Npnt. Thbs2]
KEGG	Adherens junction	3	2.4 E-3	[Fgfr1. Igf1r. Lmo7]
KEGG	Melanoma	3	4.3 E-3	[Ccnd1. Fgfr1. Igf1r]

BP = biological processes; CC= cellular components; MF= molecular functions; KEGG= Kyoto Encyclopedia of Genes and Genomes.
