# Supplementary material for: Exosomal miRNAs from Prostate Cancer Impair Osteoblast Function in Mice
Source: Int J Mol Sci. 2022 Jan 24;23(3):1285. doi: 10.3390/ijms23031285 (PMC8836054; doi:10.3390/ijms23031285)
Supplement: Supplementary file 1 [file ijms-23-01285-s001.zip › Supplementary Table S6.pdf]

Suppl. Table S6. List of all identified miRNAs in RM1-BM-EVs.

Read counts are normalized to 1 million mapped miRNAs.

Exosomal miRNAs	Reads per millions (RPM)			
	n1	n2	n3	mean
mmu-miR-21a-5p	230207.6	166507.5	148812.4	181842.5
mmu-let-7f-5p	101928	97335.78	103267.2	100843.6
mmu-miR-182-5p	70896.17	84865.52	98526.78	84762.82
mmu-miR-92a-3p	41346.73	52860.36	67280.24	53829.11
mmu-let-7a-5p	37317.44	41380.9	41770.43	40156.26
mmu-let-7i-5p	36712.52	37093.96	37042.37	36949.62
mmu-miR-183-5p	26867.9	32969.46	42946.59	34261.32
mmu-let-7c-5p	24781.44	29459.03	29968.15	28069.54
mmu-miR-30d-5p	19939.57	21907.06	20478.79	20775.14
mmu-miR-143-3p	25062.15	20306.8	16625.16	20664.7
mmu-miR-23a-3p	16772.95	20152.42	20080.99	19002.12
mmu-let-7g-5p	17901.54	18038.33	16020.45	17320.11
mmu-miR-26a-5p	15355.43	17138.55	17611.66	16701.88
mmu-miR-10b-5p	13087.58	15976.08	20014.48	16359.38
mmu-miR-30a-5p	16489.77	15190.35	14354.12	15344.75
mmu-miR-26b-5p	12894.69	12458.74	12793.7	12715.71
mmu-miR-99b-5p	8834.22	11855.04	14086.87	11592.04
mmu-miR-140-3p	9526.15	11921.87	12849.12	11432.38
mmu-miR-24-3p	11500.16	11860.8	8777.5	10712.82
mmu-miR-16-5p	10299.34	10890.74	8379.7	9856.593
mmu-miR-199a-3p	9555.7	9290.48	8340.29	9062.157
mmu-miR-199b-3p	9534.35	9253.62	8304.58	9030.85
mmu-miR-98-5p	8105.35	8416.04	10167.96	8896.45
mmu-let-7d-5p	7653.91	9519.75	8528.72	8567.46
mmu-miR-221-3p	7864.86	8956.38	8687.6	8502.947

mmu-miR-222-3p	7430.66	9035.87	9020.13	8495.553
mmu-miR-29a-3p	7172.93	9011.68	9132.2	8438.937
mmu-miR-93-5p	8566.64	9634.96	6802.04	8334.547
mmu-miR-30c-5p	7709.73	9537.03	7648.14	8298.3
mmu-let-7e-5p	7030.93	8763.98	8642.03	8145.647
mmu-miR-27b-3p	6975.12	6880.3	7181.37	7012.263
mmu-miR-25-3p	6316.84	6907.95	7619.81	6948.2
mmu-miR-27a-3p	6314.38	5904.48	6130.83	6116.563
mmu-miR-103-3p	6100.97	6478.22	5386.95	5988.713
mmu-miR-155-5p	5005.21	5742.03	5113.54	5286.927
mmu-miR-7a-5p	6388.25	4413.67	3219.36	4673.76
mmu-miR-151-3p	3703.43	4763.91	5139.4	4535.58
mmu-miR-22-3p	4896.86	5323.82	3332.67	4517.783
mmu-let-7b-5p	4007.12	4753.54	4668.94	4476.533
mmu-miR-23b-3p	3325.04	4367.59	4724.36	4138.997
mmu-miR-31-5p	4103.15	4428.65	3533.42	4021.74
mmu-miR-196b-5p	3329.97	3614.12	4010.04	3651.377
mmu-miR-191-5p	3048.43	3763.89	3661.5	3491.273
mmu-miR-15b-5p	2913	4144.08	3353.61	3470.23
mmu-miR-20a-5p	4424.09	3677.48	2182.37	3427.98
mmu-miR-125b-5p	2847.34	3221.25	3123.3	3063.963
mmu-miR-17-5p	3258.56	3361.81	2110.94	2910.437
mmu-miR-652-3p	2805.48	2843.37	2830.18	2826.343
mmu-miR-30e-5p	3808.49	2572.63	1597.37	2659.497
mmu-miR-374b-5p	2530.51	2949.36	2158.97	2546.28
mmu-miR-186-5p	2830.92	2690.14	1933.59	2484.883
mmu-miR-34c-5p	2731.61	2525.39	2076.45	2444.483
mmu-miR-320-3p	2010.13	2626.77	2464.4	2367.1
mmu-miR-151-5p	2147.2	2703.96	2213.16	2354.773
mmu-let-7j	2281.81	2443.59	2134.34	2286.58
mmu-miR-30b-5p	2200.55	2103.72	1636.78	1980.35

mmu-miR-199a-5p	1926.41	2178.61	1578.89	1894.637
mmu-miR-214-3p	1538.99	2118.7	1899.1	1852.263
mmu-miR-100-5p	1393.71	1771.92	2299.37	1821.667
mmu-miR-378a-3p	1737.62	2017.32	1642.93	1799.29
mmu-miR-125a-5p	1422.44	1748.88	1869.55	1680.29
mmu-miR-423-3p	1481.53	1593.35	1658.94	1577.94
mmu-miR-196a-5p	1423.26	1414.77	1860.92	1566.317
mmu-miR-467a-5p	1681.81	1472.38	1128.13	1427.44
mmu-miR-467b-5p	1681.81	1472.38	1128.13	1427.44
mmu-miR-361-5p	1374.83	1533.44	1307.94	1405.403
mmu-miR-34b-3p	1144.19	1438.96	1589.98	1391.043
mmu-miR-425-5p	1237.76	1535.74	1291.93	1355.143
mmu-miR-132-3p	1145.83	1366.38	1464.35	1325.52
mmu-miR-101b-3p	1716.28	958.54	703.23	1126.017
mmu-miR-148b-3p	1108.89	1111.77	955.71	1058.79
mmu-miR-145a-5p	913.54	1255.78	1001.28	1056.867
mmu-miR-669c-5p	975.93	997.71	1087.49	1020.377
mmu-miR-423-5p	955.41	942.41	964.33	954.05
mmu-miR-106b-5p	1451.17	792.64	587.47	943.76
mmu-miR-24-2-5p	964.43	960.85	833.78	919.6867
mmu-miR-532-5p	766.62	821.44	1125.67	904.5767
mmu-miR-30a-3p	752.67	937.8	975.42	888.63
mmu-miR-351-5p	791.25	884.81	977.88	884.6467
mmu-miR-96-5p	957.05	615.22	883.05	818.44
mmu-miR-466e-3p	880.71	797.25	692.15	790.0367
mmu-miR-466b-3p	879.89	796.1	692.15	789.38
mmu-miR-466c-3p	879.89	796.1	692.15	789.38
mmu-miR-466p-3p	879.89	796.1	692.15	789.38
mmu-miR-146b-5p	774.01	760.38	697.08	743.8233
mmu-miR-466a-3p	816.69	740.8	641.66	733.05
mmu-miR-322-3p	776.47	806.47	598.55	727.1633

mmu-miR-99a-5p	531.88	744.25	900.29	725.4733
mmu-miR-106b-3p	517.92	782.27	734.02	678.07
mmu-miR-467e-5p	828.18	629.04	559.14	672.12
mmu-miR-669a-5p	708.35	723.51	549.29	660.3833
mmu-miR-669p-5p	708.35	723.51	549.29	660.3833
mmu-miR-674-3p	653.35	723.51	576.38	651.08
mmu-miR-34c-3p	467.03	700.47	699.54	622.3467
mmu-miR-467a-3p	573.74	571.44	679.83	608.3367
mmu-miR-467d-3p	573.74	571.44	679.83	608.3367
mmu-miR-342-3p	454.72	629.04	650.28	578.0133
mmu-miR-28a-3p	485.09	620.98	604.71	570.26
mmu-miR-30e-3p	529.41	596.78	525.89	550.6933
mmu-miR-1839-5p	514.64	527.66	556.68	532.9933
mmu-miR-466d-3p	612.31	483.88	501.25	532.48
mmu-miR-872-5p	573.74	468.9	516.03	519.5567
mmu-miR-128-3p	494.12	563.37	447.07	501.52
mmu-miR-149-5p	375.1	556.46	534.51	488.69
mmu-miR-467c-5p	481.81	540.33	439.68	487.2733
mmu-miR-107-3p	457.18	595.63	407.65	486.82
mmu-miR-484	538.44	435.49	472.93	482.2867
mmu-miR-34a-5p	573.74	368.67	400.27	447.56
mmu-miR-142a-5p	460.47	433.19	434.75	442.8033
mmu-miR-28a-5p	363.61	468.9	415.04	415.85
mmu-miR-362-5p	346.38	422.82	460.61	409.9367
mmu-miR-3535	324.21	377.89	400.27	367.4567
mmu-miR-365-3p	314.36	450.47	337.45	367.4267
mmu-miR-129-5p	312.72	353.69	411.35	359.2533
mmu-miR-501-3p	361.97	374.43	341.15	359.1833
mmu-miR-421-3p	302.87	384.8	381.79	356.4867
mmu-miR-101a-3p	600.82	207.38	254.94	354.38
mmu-miR-450a-5p	439.95	308.76	301.74	350.15

mmu-miR-99b-3p	299.59	396.32	338.69	344.8667
mmu-miR-18a-5p	503.15	290.33	190.9	328.1267
mmu-miR-126a-3p	358.69	291.48	285.73	311.9667
mmu-miR-1983	282.35	330.65	320.21	311.07
mmu-miR-130a-3p	263.48	329.5	285.73	292.9033
mmu-miR-19b-3p	548.29	263.83	60.35	290.8233
mmu-miR-221-5p	256.09	296.09	314.05	288.7433
mmu-miR-674-5p	265.12	322.59	261.1	282.9367
mmu-miR-669a-3p	414.5	191.25	238.93	281.56
mmu-miR-669o-3p	414.5	191.25	238.93	281.56
mmu-miR-467d-5p	274.97	322.59	232.77	276.7767
mmu-miR-298-5p	239.67	351.39	232.77	274.61
mmu-miR-744-5p	275.79	300.7	229.07	268.52
mmu-miR-6240	219.97	321.43	256.17	265.8567
mmu-miR-140-5p	283.17	254.61	240.16	259.3133
mmu-miR-9-5p	285.64	214.29	274.64	258.19
mmu-miR-324-5p	252.8	262.68	253.71	256.3967
mmu-miR-361-3p	209.3	256.92	274.64	246.9533
mmu-miR-322-5p	264.3	254.61	221.69	246.8667
mmu-let-7a-1-3p	283.17	194.7	259.86	245.91
mmu-let-7c-2-3p	283.17	194.7	259.86	245.91
mmu-miR-212-3p	228.18	307.61	195.82	243.87
mmu-miR-345-3p	206.02	240.79	209.37	218.7267
mmu-miR-503-5p	247.88	197.01	169.96	204.95
mmu-miR-185-5p	175.65	231.57	206.91	204.71
mmu-miR-350-3p	174.01	214.29	192.13	193.4767
mmu-miR-1198-5p	164.98	163.6	250.01	192.8633
mmu-let-7d-3p	174.83	190.1	213.06	192.6633
mmu-miR-450b-3p	172.37	206.22	198.29	192.2933
mmu-miR-125b-1-3p	156.77	209.68	198.29	188.2467
mmu-miR-378c	179.75	199.31	179.81	186.29

mmu-miR-206-3p	176.47	175.12	205.67	185.7533
mmu-miR-15a-5p	259.37	183.18	113.31	185.2867
mmu-miR-15b-3p	161.7	184.33	199.52	181.85
mmu-miR-194-5p	220.79	210.83	98.53	176.7167
mmu-miR-218-5p	195.35	167.05	167.5	176.6333
mmu-miR-671-5p	174.83	246.55	105.92	175.7667
mmu-miR-542-3p	286.46	149.77	78.82	171.6833
mmu-miR-212-5p	124.76	156.68	224.15	168.53
mmu-miR-5099	193.71	188.94	117	166.55
mmu-miR-192-5p	175.65	162.45	160.11	166.07
mmu-miR-22-5p	165.8	175.12	129.32	156.7467
mmu-miR-152-3p	176.47	153.23	140.4	156.7
mmu-miR-129-2-3p	116.55	148.62	192.13	152.4333
mmu-miR-130b-3p	119.84	158.99	167.5	148.7767
mmu-miR-574-5p	124.76	129.03	188.43	147.4067
mmu-miR-199b-5p	183.86	137.1	120.7	147.22
mmu-miR-326-3p	116.55	120.97	185.97	141.1633
mmu-miR-486b-5p	85.36	129.03	197.05	137.1467
mmu-miR-574-3p	121.48	135.95	152.72	136.7167
mmu-miR-486a-5p	84.54	124.43	197.05	135.34
mmu-miR-183-3p	137.07	142.86	121.93	133.9533
mmu-miR-324-3p	96.85	162.45	129.32	129.54
mmu-miR-181a-5p	89.47	137.1	142.86	123.1433
mmu-miR-130b-5p	109.17	139.4	119.46	122.6767
mmu-miR-210-3p	92.75	153.23	118.23	121.4033
mmu-miR-328-3p	91.93	101.38	149.02	114.11
mmu-miR-339-5p	102.6	111.75	118.23	110.86
mmu-miR-98-3p	119.02	97.93	107.15	108.0333
mmu-miR-466f-3p	110.81	105.99	99.76	105.52
mmu-miR-214-5p	105.06	115.21	87.44	102.57
mmu-miR-145a-3p	97.67	88.71	117	101.1267

mmu-miR-190b-5p	130.51	103.69	68.97	101.0567
mmu-miR-5121	67.31	134.79	99.76	100.62
mmu-miR-297a-3p	100.14	103.69	97.3	100.3767
mmu-miR-297b-3p	100.14	103.69	97.3	100.3767
mmu-miR-297c-3p	100.14	103.69	97.3	100.3767
mmu-miR-17-3p	88.65	100.23	112.07	100.3167
mmu-miR-669l-5p	105.06	105.99	88.67	99.90667
mmu-miR-1981-5p	75.51	101.38	114.54	97.14333
mmu-miR-132-5p	91.11	80.65	108.38	93.38
mmu-miR-664-3p	91.93	101.38	80.05	91.12
mmu-miR-672-5p	61.56	112.91	89.91	88.12667
mmu-miR-669o-5p	96.03	57.6	100.99	84.87333
mmu-miR-125a-3p	85.36	80.65	86.21	84.07333
mmu-let-7i-3p	76.33	117.51	57.88	83.90667
mmu-miR-872-3p	104.24	73.73	59.12	79.03
mmu-miR-690	63.2	80.65	89.91	77.92
mmu-miR-181b-5p	69.77	81.8	73.9	75.15667
mmu-miR-18b-5p	112.45	65.67	35.72	71.28
mmu-miR-29c-3p	78.8	65.67	67.74	70.73667
mmu-miR-708-5p	87.83	63.37	57.88	69.69333
mmu-miR-466g	80.44	62.21	65.27	69.30667
mmu-miR-671-3p	68.95	70.28	66.51	68.58
mmu-miR-345-5p	60.74	78.34	64.04	67.70667
mmu-miR-10a-5p	62.38	71.43	68.97	67.59333
mmu-miR-193b-3p	41.86	65.67	93.6	67.04333
mmu-miR-30c-2-3p	62.38	66.82	71.43	66.87667
mmu-miR-18a-3p	64.02	73.73	61.58	66.44333
mmu-miR-31-3p	59.1	76.04	61.58	65.57333
mmu-miR-582-3p	47.61	85.25	61.58	64.81333
mmu-miR-331-3p	53.35	59.91	80.05	64.43667
mmu-miR-29b-3p	73.05	57.6	59.12	63.25667

mmu-miR-1843a-5p	62.38	44.93	80.05	62.45333
mmu-miR-222-5p	44.32	62.21	77.59	61.37333
mmu-miR-1a-3p	54.99	64.52	59.12	59.54333
mmu-miR-450b-5p	88.65	35.71	49.26	57.87333
mmu-miR-500-3p	53.35	51.84	67.74	57.64333
mmu-miR-3068-3p	48.43	66.82	55.42	56.89
mmu-miR-10b-3p	64.02	61.06	41.87	55.65
mmu-miR-1945	45.96	73.73	45.57	55.08667
mmu-miR-1843b-5p	51.71	40.32	67.74	53.25667
mmu-miR-700-3p	31.19	54.15	73.9	53.08
mmu-miR-9-3p	59.92	63.37	35.72	53.00333
mmu-miR-2137	26.27	93.32	36.95	52.18
mmu-miR-615-3p	42.68	42.63	66.51	50.60667
mmu-miR-92b-3p	33.65	47.24	62.81	47.9
mmu-miR-93-3p	41.86	36.87	64.04	47.59
mmu-miR-296-5p	30.37	51.84	60.35	47.52
mmu-miR-7688-5p	33.65	35.71	68.97	46.11
mmu-miR-664-5p	56.63	44.93	33.25	44.93667
mmu-miR-378a-5p	41.86	51.84	40.64	44.78
mmu-miR-877-5p	52.53	50.69	30.79	44.67
mmu-miR-501-5p	41.86	61.06	30.79	44.57
mmu-let-7e-3p	49.25	50.69	33.25	44.39667
mmu-miR-122-5p	36.94	73.73	19.71	43.46
mmu-miR-301a-5p	50.89	43.78	34.48	43.05
mmu-miR-16-2-3p	61.56	39.17	25.86	42.19667
mmu-miR-1187	37.76	29.95	57.88	41.86333
mmu-miR-425-3p	41.04	42.63	41.87	41.84667
mmu-miR-362-3p	48.43	33.41	39.41	40.41667
mmu-miR-1981-3p	34.47	43.78	41.87	40.04
mmu-miR-342-5p	20.52	43.78	52.96	39.08667
mmu-miR-142a-3p	82.08	16.13	13.55	37.25333

mmu-miR-335-5p	53.35	38.02	16.01	35.79333
mmu-miR-296-3p	26.27	57.6	23.4	35.75667
mmu-miR-335-3p	31.19	42.63	33.25	35.69
mmu-miR-1191a	31.19	34.56	40.64	35.46333
mmu-miR-193a-5p	29.55	48.39	28.33	35.42333
mmu-miR-374c-5p	28.73	50.69	23.4	34.27333
mmu-miR-350-5p	27.91	43.78	30.79	34.16
mmu-miR-466h-3p	36.94	33.41	30.79	33.71333
mmu-miR-3968	18.88	38.02	43.11	33.33667
mmu-miR-669h-5p	19.7	39.17	40.64	33.17
mmu-miR-7a-1-3p	22.16	46.08	30.79	33.01
mmu-miR-181d-5p	27.09	41.48	28.33	32.3
mmu-miR-1949	36.11	39.17	17.24	30.84
mmu-miR-301a-3p	55.81	26.5	9.85	30.72
mmu-miR-466n-3p	40.22	25.35	22.17	29.24667
mmu-miR-19a-3p	57.46	5.76	19.71	27.64333
mmu-miR-126a-5p	22.98	31.11	28.33	27.47333
mmu-miR-669f-5p	29.55	33.41	18.47	27.14333
mmu-miR-669b-5p	47.61	21.89	11.08	26.86
mmu-miR-339-3p	20.52	34.56	23.4	26.16
mmu-miR-669d-5p	44.32	18.43	14.78	25.84333
mmu-miR-700-5p	32.01	29.95	14.78	25.58
mmu-miR-29b-1-5p	18.88	27.65	29.56	25.36333
mmu-miR-708-3p	27.91	18.43	29.56	25.3
mmu-miR-215-5p	13.13	18.43	43.11	24.89
mmu-miR-27b-5p	10.67	23.04	39.41	24.37333
mmu-miR-505-3p	22.98	29.95	19.71	24.21333
mmu-let-7f-1-3p	20.52	21.89	29.56	23.99
mmu-miR-8114	24.62	19.59	27.09	23.76667
mmu-miR-466i-3p	32.83	13.83	24.63	23.76333
mmu-miR-3066-5p	36.11	12.67	22.17	23.65

mmu-miR-877-3p	16.42	35.71	18.47	23.53333
mmu-miR-138-5p	20.52	26.5	23.4	23.47333
mmu-miR-34b-5p	42.68	19.59	7.39	23.22
mmu-miR-331-5p	18.88	20.74	29.56	23.06
mmu-miR-532-3p	15.6	28.8	24.63	23.01
mmu-miR-3473a	16.42	25.35	27.09	22.95333
mmu-miR-125b-2-3p	22.98	20.74	24.63	22.78333
mmu-miR-200b-3p	22.16	25.35	18.47	21.99333
mmu-miR-1943-5p	13.95	32.26	19.71	21.97333
mmu-miR-143-5p	18.88	35.71	11.08	21.89
mmu-miR-3102-3p	11.49	24.19	29.56	21.74667
mmu-miR-466c-5p	22.16	21.89	19.71	21.25333
mmu-miR-703	17.24	29.95	16.01	21.06667
mmu-miR-450a-1-3p	33.65	9.22	18.47	20.44667
mmu-miR-503-3p	14.77	34.56	11.08	20.13667
mmu-miR-375-3p	6.57	10.37	40.64	19.19333
mmu-miR-32-5p	44.32	6.91	6.16	19.13
mmu-miR-28c	21.34	14.98	20.94	19.08667
mmu-miR-8112	20.52	2.3	30.79	17.87
mmu-miR-5113	21.34	19.59	12.32	17.75
mmu-miR-148b-5p	13.13	23.04	16.01	17.39333
mmu-miR-21a-3p	23.8	21.89	4.93	16.87333
mmu-let-7b-3p	13.95	9.22	24.63	15.93333
mmu-miR-191-3p	8.21	16.13	23.4	15.91333
mmu-miR-1249-3p	16.42	16.13	14.78	15.77667
mmu-miR-378d	12.31	16.13	18.47	15.63667
mmu-miR-1306-3p	13.95	12.67	19.71	15.44333
mmu-miR-3068-5p	14.77	16.13	12.32	14.40667
mmu-miR-466i-5p	10.67	6.91	24.63	14.07
mmu-miR-1955-5p	13.95	3.46	24.63	14.01333
mmu-miR-378b	20.52	13.83	4.93	13.09333

mmu-miR-195a-5p	13.95	8.06	17.24	13.08333
mmu-miR-652-5p	16.42	14.98	7.39	12.93
mmu-miR-542-5p	11.49	13.83	12.32	12.54667
mmu-miR-181c-5p	16.42	16.13	4.93	12.49333
mmu-miR-139-5p	13.13	18.43	4.93	12.16333
mmu-miR-24-1-5p	18.06	6.91	11.08	12.01667
mmu-miR-1291	7.39	13.83	14.78	12
mmu-miR-669e-5p	12.31	13.83	8.62	11.58667
mmu-miR-467e-3p	18.06	9.22	6.16	11.14667
mmu-miR-1306-5p	12.31	10.37	9.85	10.84333
mmu-miR-669f-3p	20.52	9.22	2.46	10.73333
mmu-miR-106a-5p	13.13	10.37	8.62	10.70667
mmu-miR-466m-3p	4.1	8.06	19.71	10.62333
mmu-miR-29c-5p	9.03	10.37	11.08	10.16
mmu-miR-3074-5p	11.49	12.67	6.16	10.10667
mmu-miR-25-5p	14.77	6.91	8.62	10.1
mmu-miR-669k-5p	3.28	4.61	22.17	10.02
mmu-miR-1194	4.92	13.83	11.08	9.943333
mmu-miR-3082-3p	9.03	6.91	13.55	9.83
mmu-miR-146a-5p	8.21	13.83	7.39	9.81
mmu-miR-1964-3p	4.1	12.67	12.32	9.696667
mmu-miR-3473b	11.49	12.67	4.93	9.696667
mmu-miR-3057-5p	9.85	8.06	11.08	9.663333
mmu-miR-3061-3p	9.85	10.37	7.39	9.203333
mmu-miR-3473d	5.75	12.67	8.62	9.013333
mmu-miR-188-5p	7.39	9.22	9.85	8.82
mmu-miR-582-5p	12.31	10.37	3.69	8.79
mmu-miR-3064-5p	15.6	8.06	2.46	8.706667
mmu-miR-33-3p	11.49	8.06	6.16	8.57
mmu-miR-3074-1-3p	7.39	5.76	12.32	8.49
mmu-miR-449a-5p	10.67	4.61	9.85	8.376667

mmu-miR-379-5p	8.21	6.91	9.85	8.323333
mmu-miR-1839-3p	8.21	8.06	8.62	8.296667
mmu-miR-101c	11.49	5.76	7.39	8.213333
mmu-miR-203-3p	9.03	5.76	9.85	8.213333
mmu-miR-301b-3p	9.03	14.98	0	8.003333
mmu-miR-148a-3p	8.21	3.46	12.32	7.996667
mmu-miR-466h-5p	9.85	11.52	2.46	7.943333
mmu-miR-541-5p	4.1	5.76	13.55	7.803333
mmu-miR-147-5p	7.39	6.91	8.62	7.64
mmu-miR-20a-3p	15.6	6.91	0	7.503333
mmu-miR-669p-3p	10.67	8.06	3.69	7.473333
mmu-miR-466b-5p	17.24	1.15	3.69	7.36
mmu-miR-466o-5p	17.24	1.15	3.69	7.36
mmu-miR-5123	9.03	8.06	4.93	7.34
mmu-miR-1948-3p	9.85	2.3	9.85	7.333333
mmu-miR-34a-3p	5.75	4.61	11.08	7.146667
mmu-miR-16-1-3p	8.21	6.91	6.16	7.093333
mmu-miR-3473e	9.03	9.22	2.46	6.903333
mmu-miR-451a	8.21	10.37	1.23	6.603333
mmu-miR-5126	4.1	6.91	8.62	6.543333
mmu-miR-3086-5p	9.85	2.3	7.39	6.513333
mmu-miR-467c-3p	9.85	5.76	3.69	6.433333
mmu-miR-669a-3-3p	9.03	2.3	7.39	6.24
mmu-miR-150-5p	4.1	6.91	7.39	6.133333
mmu-miR-27a-5p	2.46	1.15	14.78	6.13
mmu-miR-193a-3p	4.92	2.3	11.08	6.1
mmu-miR-182-3p	2.46	3.46	12.32	6.08
mmu-miR-21b	7.39	5.76	4.93	6.026667
mmu-miR-129-1-3p	5.75	4.61	7.39	5.916667
mmu-miR-3102-3p.2-3p	3.28	8.06	6.16	5.833333

mmu-miR-744-3p	4.1	3.46	9.85	5.803333
mmu-miR-7061-5p	2.46	0	14.78	5.746667
mmu-miR-351-3p	6.57	6.91	3.69	5.723333
mmu-miR-491-3p	4.92	4.61	7.39	5.64
mmu-miR-500-5p	2.46	4.61	9.85	5.64
mmu-miR-3089-3p	7.39	6.91	2.46	5.586667
mmu-miR-32-3p	5.75	3.46	7.39	5.533333
mmu-miR-340-5p	4.1	1.15	11.08	5.443333
mmu-miR-29b-2-5p	0.82	8.06	7.39	5.423333
mmu-miR-670-5p	0	0	16.01	5.336667
mmu-let-7f-2-3p	5.75	4.61	4.93	5.096667
mmu-miR-466k	13.95	1.15	0	5.033333
mmu-miR-1191b-5p	4.1	3.46	7.39	4.983333
mmu-miR-29a-5p	9.03	4.61	1.23	4.956667
mmu-miR-449c-5p	4.1	5.76	4.93	4.93
mmu-miR-219a-1-3p	6.57	8.06	0	4.876667
mmu-miR-1b-5p	7.39	3.46	3.69	4.846667
mmu-miR-152-5p	8.21	0	6.16	4.79
mmu-miR-3076-3p	4.92	8.06	1.23	4.736667
mmu-miR-6238	5.75	2.3	6.16	4.736667
mmu-miR-1843b-3p	2.46	8.06	3.69	4.736667
mmu-miR-669m-3p	5.75	4.61	3.69	4.683333
mmu-miR-760-3p	5.75	5.76	2.46	4.656667
mmu-miR-6900-3p	1.64	5.76	6.16	4.52
mmu-miR-466e-5p	9.85	0	3.69	4.513333
mmu-miR-702-3p	2.46	5.76	4.93	4.383333
mmu-miR-6516-3p	10.67	0	2.46	4.376667
mmu-miR-7021-5p	3.28	3.46	6.16	4.3
mmu-miR-7669-5p	3.28	3.46	6.16	4.3
mmu-miR-130a-5p	5.75	4.61	2.46	4.273333
mmu-miR-3470b	5.75	5.76	1.23	4.246667

mmu-miR-466m-5p	6.57	3.46	2.46	4.163333
mmu-miR-103-2-5p	1.64	3.46	7.39	4.163333
mmu-miR-1195	4.1	5.76	2.46	4.106667
mmu-miR-6975-3p	0	2.3	9.85	4.05
mmu-miR-190a-5p	7.39	3.46	1.23	4.026667
mmu-miR-3965	4.92	4.61	2.46	3.996667
mmu-miR-497a-5p	4.92	4.61	2.46	3.996667
mmu-miR-7b-5p	5.75	3.46	2.46	3.89
mmu-miR-26a-2-3p	3.28	3.46	4.93	3.89
mmu-miR-297a-5p	9.03	0	2.46	3.83
mmu-miR-338-5p	1.64	1.15	8.62	3.803333
mmu-miR-669m-5p	6.57	2.3	2.46	3.776667
mmu-miR-147-3p	1.64	3.46	6.16	3.753333
mmu-miR-3058-3p	1.64	5.76	3.69	3.696667
mmu-miR-3084-3p	1.64	5.76	3.69	3.696667
mmu-miR-3060-3p	4.1	6.91	0	3.67
mmu-miR-193b-5p	0	1.15	9.85	3.666667
mmu-miR-3084-5p	4.92	2.3	3.69	3.636667
mmu-miR-7070-3p	0	6.91	3.69	3.533333
mmu-miR-6239	5.75	1.15	3.69	3.53
mmu-miR-3470a	2.46	8.06	0	3.506667
mmu-miR-1934-5p	0.82	2.3	7.39	3.503333
mmu-miR-3062-5p	5.75	3.46	1.23	3.48
mmu-miR-467h	5.75	4.61	0	3.453333
mmu-miR-30c-1-3p	6.57	0	3.69	3.42
mmu-miR-96-3p	4.1	1.15	4.93	3.393333
mmu-miR-20b-5p	6.57	2.3	1.23	3.366667
mmu-miR-3970	4.1	3.46	2.46	3.34
mmu-miR-1930-5p	2.46	0	7.39	3.283333
mmu-miR-23b-5p	1.64	6.91	1.23	3.26
mmu-miR-7033-5p	0	2.3	7.39	3.23

mmu-miR-7235-3p	2.46	3.46	3.69	3.203333
mmu-miR-466p-5p	4.92	4.61	0	3.176667
mmu-miR-467g	2.46	5.76	1.23	3.15
mmu-miR-344-3p	0.82	0	8.62	3.146667
mmu-miR-505-5p	2.46	6.91	0	3.123333
mmu-miR-30d-3p	3.28	2.3	3.69	3.09
mmu-miR-669b-3p	3.28	4.61	1.23	3.04
mmu-miR-186-3p	1.64	2.3	4.93	2.956667
mmu-miR-365-2-5p	4.1	2.3	2.46	2.953333
mmu-miR-466o-3p	1.64	3.46	3.69	2.93
mmu-miR-5114	4.1	4.61	0	2.903333
mmu-miR-669g	0	0	8.62	2.873333
mmu-miR-219a-5p	0	1.15	7.39	2.846667
mmu-miR-6951-5p	0	1.15	7.39	2.846667
mmu-miR-200c-3p	4.92	2.3	1.23	2.816667
mmu-miR-6994-3p	0	3.46	4.93	2.796667
mmu-miR-7680-3p	2.46	3.46	2.46	2.793333
mmu-miR-184-3p	0	4.61	3.69	2.766667
mmu-miR-467b-3p	0.82	0	7.39	2.736667
mmu-miR-466j	5.75	1.15	1.23	2.71
mmu-miR-466f	3.28	1.15	3.69	2.706667
mmu-miR-6944-3p	0	8.06	0	2.686667
mmu-miR-26b-3p	3.28	2.3	2.46	2.68
mmu-miR-3075-5p	3.28	2.3	2.46	2.68
mmu-miR-187-3p	0.82	4.61	2.46	2.63
mmu-miR-669e-3p	3.28	4.61	0	2.63
mmu-miR-429-3p	1.64	0	6.16	2.6
mmu-miR-6418-3p	1.64	0	6.16	2.6
mmu-miR-8094	1.64	0	6.16	2.6
mmu-miR-3473h-5p	1.64	1.15	4.93	2.573333
mmu-miR-3962	4.1	1.15	2.46	2.57

mmu-miR-30b-3p	4.1	2.3	1.23	2.543333
mmu-miR-7117-5p	0	0	7.39	2.463333
mmu-miR-19b-1-5p	4.92	0	2.46	2.46
mmu-miR-7091-5p	2.46	2.3	2.46	2.406667
mmu-miR-344d-3p	0	3.46	3.69	2.383333
mmu-miR-6932-5p	0	3.46	3.69	2.383333
mmu-miR-6967-3p	2.46	3.46	1.23	2.383333
mmu-miR-6516-5p	3.28	2.3	1.23	2.27
mmu-miR-1931	0.82	4.61	1.23	2.22
mmu-miR-5128	0.82	4.61	1.23	2.22
mmu-miR-7018-3p	1.64	0	4.93	2.19
mmu-miR-6960-5p	4.1	0	2.46	2.186667
mmu-miR-491-5p	4.1	1.15	1.23	2.16
mmu-miR-15a-3p	1.64	2.3	2.46	2.133333
mmu-miR-210-5p	1.64	2.3	2.46	2.133333
mmu-miR-218-1-3p	1.64	2.3	2.46	2.133333
mmu-miR-470-5p	4.1	2.3	0	2.133333
mmu-miR-301b-5p	1.64	4.61	0	2.083333
mmu-miR-144-5p	0	0	6.16	2.053333
mmu-miR-712-3p	0	0	6.16	2.053333
mmu-miR-421-5p	2.46	0	3.69	2.05
mmu-miR-190b-3p	0	1.15	4.93	2.026667
mmu-miR-298-3p	0	1.15	4.93	2.026667
mmu-miR-499-5p	4.92	1.15	0	2.023333
mmu-miR-194-2-3p	0	2.3	3.69	1.996667
mmu-miR-411-3p	2.46	2.3	1.23	1.996667
mmu-miR-677-5p	2.46	2.3	1.23	1.996667
mmu-miR-6896-5p	2.46	2.3	1.23	1.996667
mmu-miR-1970c-5p	2.46	3.46	0	1.973333
mmu-miR-26a-1-3p	2.46	3.46	0	1.973333
mmu-miR-5132-5p	0	3.46	2.46	1.973333

mmu-miR-465c-5p	0	4.61	1.23	1.946667
mmu-miR-6236	0	4.61	1.23	1.946667
mmu-miR-7654-5p	0	5.76	0	1.92
mmu-miR-466f-5p	0.82	1.15	3.69	1.886667
mmu-miR-466l-3p	0.82	1.15	3.69	1.886667
mmu-miR-5100	3.28	1.15	1.23	1.886667
mmu-let-7g-3p	3.28	2.3	0	1.86
mmu-miR-145b	0.82	2.3	2.46	1.86
mmu-miR-1843a-3p	3.28	2.3	0	1.86
mmu-miR-1982-3p	0.82	2.3	2.46	1.86
mmu-miR-3061-5p	3.28	2.3	0	1.86
mmu-miR-3963	0.82	2.3	2.46	1.86
mmu-miR-6967-5p	3.28	2.3	0	1.86
mmu-miR-7224-3p	1.64	0	3.69	1.776667
mmu-miR-155-3p	1.64	1.15	2.46	1.75
mmu-miR-181a-2-3p	1.64	3.46	0	1.7
mmu-miR-1929-5p	1.64	3.46	0	1.7
mmu-miR-1950	0	0	4.93	1.643333
mmu-miR-7042-5p	0	0	4.93	1.643333
mmu-miR-7647-5p	0	0	4.93	1.643333
mmu-miR-7667-3p	0	0	4.93	1.643333
mmu-miR-7672-5p	0	0	4.93	1.643333
mmu-miR-23a-5p	2.46	2.3	0	1.586667
mmu-miR-92a-1-5p	0	3.46	1.23	1.563333
mmu-miR-219c-5p	0	4.61	0	1.536667
mmu-miR-3112-5p	0	4.61	0	1.536667
mmu-miR-7118-3p	0	4.61	0	1.536667
mmu-miR-3058-5p	0.82	0	3.69	1.503333
mmu-miR-3098-3p	3.28	0	1.23	1.503333
mmu-miR-7669-3p	0.82	0	3.69	1.503333
mmu-miR-1933-3p	0.82	1.15	2.46	1.476667

mmu-miR-21c	3.28	1.15	0	1.476667
mmu-miR-409-3p	3.28	1.15	0	1.476667
mmu-miR-181a-1-3p	0.82	3.46	0	1.426667
mmu-miR-3063-3p	0.82	3.46	0	1.426667
mmu-miR-466a-5p	0.82	3.46	0	1.426667
mmu-miR-7664-3p	0.82	3.46	0	1.426667
mmu-miR-7667-5p	1.64	0	2.46	1.366667
mmu-miR-12194-3p	1.64	2.3	0	1.313333
mmu-miR-1955-3p	1.64	2.3	0	1.313333
mmu-miR-1969	1.64	2.3	0	1.313333
mmu-miR-28b	1.64	2.3	0	1.313333
mmu-miR-6900-5p	1.64	2.3	0	1.313333
mmu-miR-881-3p	1.64	2.3	0	1.313333
mmu-miR-12196-3p	0	0	3.69	1.23
mmu-miR-135a-2-3p	0	0	3.69	1.23
mmu-miR-154-3p	0	0	3.69	1.23
mmu-miR-1968-5p	0	0	3.69	1.23
mmu-miR-3101-5p	0	0	3.69	1.23
mmu-miR-6538	0	0	3.69	1.23
mmu-miR-677-3p	0	0	3.69	1.23
mmu-miR-6952-5p	0	0	3.69	1.23
mmu-miR-7092-3p	0	0	3.69	1.23
mmu-miR-8105	2.46	0	1.23	1.23
mmu-miR-1941-5p	0	1.15	2.46	1.203333
mmu-miR-376a-5p	2.46	1.15	0	1.203333
mmu-miR-494-3p	0	1.15	2.46	1.203333
mmu-miR-7070-5p	0	1.15	2.46	1.203333
mmu-miR-7075-3p	0	1.15	2.46	1.203333
mmu-miR-7689-3p	2.46	1.15	0	1.203333
mmu-miR-5615-5p	0	2.3	1.23	1.176667
mmu-miR-6899-3p	0	2.3	1.23	1.176667

mmu-miR-6939-3p	0	2.3	1.23	1.176667
mmu-miR-7663-3p	0	2.3	1.23	1.176667
mmu-miR-100-3p	0	3.46	0	1.153333
mmu-miR-12189-3p	0	3.46	0	1.153333
mmu-miR-138-1-3p	0	3.46	0	1.153333
mmu-miR-3544-5p	0	3.46	0	1.153333
mmu-miR-6970-5p	0	3.46	0	1.153333
mmu-miR-6998-3p	0	3.46	0	1.153333
mmu-miR-7068-3p	0	3.46	0	1.153333
mmu-miR-7658-5p	0	3.46	0	1.153333
mmu-miR-135b-5p	0.82	0	2.46	1.093333
mmu-miR-138-2-3p	0.82	0	2.46	1.093333
mmu-miR-1934-3p	0.82	0	2.46	1.093333
mmu-miR-215-3p	0.82	0	2.46	1.093333
mmu-miR-3066-3p	0.82	0	2.46	1.093333
mmu-miR-3095-3p	0.82	0	2.46	1.093333
mmu-miR-6911-3p	0.82	0	2.46	1.093333
mmu-let-7c-1-3p	0.82	1.15	1.23	1.066667
mmu-miR-5132-3p	0.82	1.15	1.23	1.066667
mmu-miR-450a-2-3p	0.82	2.3	0	1.04
mmu-miR-871-3p	1.64	0	1.23	0.956667
mmu-miR-1941-3p	1.64	1.15	0	0.93
mmu-miR-3102-5p.2-5p	1.64	1.15	0	0.93
mmu-miR-369-3p	1.64	1.15	0	0.93
mmu-miR-706	1.64	1.15	0	0.93
mmu-miR-7061-3p	1.64	1.15	0	0.93
mmu-miR-133a-3p	0	0	2.46	0.82
mmu-miR-1936	0	0	2.46	0.82
mmu-miR-196b-3p	0	0	2.46	0.82
mmu-miR-3069-5p	0	0	2.46	0.82

mmu-miR-487b-3p	0	0	2.46	0.82
mmu-miR-676-3p	0	0	2.46	0.82
mmu-miR-7016-3p	0	0	2.46	0.82
mmu-miR-7219-3p	0	0	2.46	0.82
mmu-miR-185-3p	0	2.3	0	0.766667
mmu-miR-1895	0	2.3	0	0.766667
mmu-miR-1947-5p	0	2.3	0	0.766667
mmu-miR-223-3p	0	2.3	0	0.766667
mmu-miR-3094-3p	0	2.3	0	0.766667
mmu-miR-3103-5p	0	2.3	0	0.766667
mmu-miR-382-3p	0	2.3	0	0.766667
mmu-miR-493-5p	0	2.3	0	0.766667
mmu-miR-6911-5p	0	2.3	0	0.766667
mmu-miR-6940-3p	0	2.3	0	0.766667
mmu-miR-6942-5p	0	2.3	0	0.766667
mmu-miR-6948-5p	0	2.3	0	0.766667
mmu-miR-6977-3p	0	2.3	0	0.766667
mmu-miR-6990-5p	0	2.3	0	0.766667
mmu-miR-702-5p	0	2.3	0	0.766667
mmu-miR-7679-3p	0	2.3	0	0.766667
mmu-miR-1198-3p	0.82	0	1.23	0.683333
mmu-miR-5709-5p	0.82	0	1.23	0.683333
mmu-miR-6988-3p	0.82	0	1.23	0.683333
mmu-miR-7660-3p	0.82	0	1.23	0.683333
mmu-miR-374c-3p	0.82	1.15	0	0.656667
mmu-miR-6966-5p	0.82	1.15	0	0.656667
mmu-miR-7025-3p	0.82	1.15	0	0.656667
mmu-miR-709	0.82	1.15	0	0.656667
mmu-miR-7240-5p	0.82	1.15	0	0.656667
mmu-miR-142b	0	0	1.23	0.41
mmu-miR-1971	0	0	1.23	0.41

mmu-miR-3087-3p	0	0	1.23	0.41
mmu-miR-3102-5p	0	0	1.23	0.41
mmu-miR-340-3p	0	0	1.23	0.41
mmu-miR-3473f	0	0	1.23	0.41
mmu-miR-3474	0	0	1.23	0.41
mmu-miR-3967	0	0	1.23	0.41
mmu-miR-6546-5p	0	0	1.23	0.41
mmu-miR-6937-5p	0	0	1.23	0.41
mmu-miR-6968-3p	0	0	1.23	0.41
mmu-miR-7016-5p	0	0	1.23	0.41
mmu-miR-7656-5p	0	0	1.23	0.41
mmu-miR-7682-3p	0	0	1.23	0.41
mmu-let-7k	0	1.15	0	0.383333
mmu-miR-12201-5p	0	1.15	0	0.383333
mmu-miR-12202-3p	0	1.15	0	0.383333
mmu-miR-154-5p	0	1.15	0	0.383333
mmu-miR-205-5p	0	1.15	0	0.383333
mmu-miR-299a-3p	0	1.15	0	0.383333
mmu-miR-3057-3p	0	1.15	0	0.383333
mmu-miR-3097-5p	0	1.15	0	0.383333
mmu-miR-3108-5p	0	1.15	0	0.383333
mmu-miR-3109-5p	0	1.15	0	0.383333
mmu-miR-3110-5p	0	1.15	0	0.383333
mmu-miR-329-5p	0	1.15	0	0.383333
mmu-miR-3473g	0	1.15	0	0.383333
mmu-miR-465a-3p	0	1.15	0	0.383333
mmu-miR-465b-3p	0	1.15	0	0.383333
mmu-miR-465c-3p	0	1.15	0	0.383333
mmu-miR-5124a	0	1.15	0	0.383333
mmu-miR-5129-3p	0	1.15	0	0.383333
mmu-miR-5134-3p	0	1.15	0	0.383333

mmu-miR-6897-5p	0	1.15	0	0.383333
mmu-miR-6916-5p	0	1.15	0	0.383333
mmu-miR-6935-3p	0	1.15	0	0.383333
mmu-miR-6935-5p	0	1.15	0	0.383333
mmu-miR-6945-3p	0	1.15	0	0.383333
mmu-miR-6946-3p	0	1.15	0	0.383333
mmu-miR-6963-5p	0	1.15	0	0.383333
mmu-miR-6996-3p	0	1.15	0	0.383333
mmu-miR-6997-3p	0	1.15	0	0.383333
mmu-miR-7002-3p	0	1.15	0	0.383333
mmu-miR-7008-5p	0	1.15	0	0.383333
mmu-miR-7014-5p	0	1.15	0	0.383333
mmu-miR-7034-5p	0	1.15	0	0.383333
mmu-miR-7065-3p	0	1.15	0	0.383333
mmu-miR-7068-5p	0	1.15	0	0.383333
mmu-miR-7069-5p	0	1.15	0	0.383333
mmu-miR-741-3p	0	1.15	0	0.383333
mmu-miR-7655-5p	0	1.15	0	0.383333
mmu-miR-7658-3p	0	1.15	0	0.383333
mmu-miR-7666-3p	0	1.15	0	0.383333
mmu-miR-8098	0	1.15	0	0.383333
mmu-miR-8106	0	1.15	0	0.383333
mmu-miR-873a-5p	0	1.15	0	0.383333
